# Supplementary material for: Iron-coated Komodo dragon teeth and the complex dental enamel of carnivorous reptiles
Source: Nat Ecol Evol. 2024 Jul 24;8(9):1711–22. doi: 10.1038/s41559-024-02477-7 (PMC11383799; doi:10.1038/s41559-024-02477-7)
Supplement: Supplementary file 1 — Supplementary Figs. 1–26 and Tables 1–5. [file 41559_2024_2477_MOESM1_ESM.pdf]

nature ecology & evolution

Supplementary information https://doi.org/10.1038/s41559-024-02477-7

Iron-coated Komodo dragon teeth and the complex dental enamel of carnivorous reptiles

In the format provided by the authors and unedited

Table of Contents

Page 3 Supplementary Figure 1. **Pigmented cutting edges and tooth tips in museum specimens of *Varanus komodoensis***

Page 4 Supplementary Figure 2. **Additional synchrotron-based X-Ray MicroFluorescence (S-µXRF) and Scanning Electron Energy-Dispersive x-ray Spectroscopy (SEM-EDS) elemental maps for *Varanus komodoensis* teeth.**

Page 5 Supplementary Figure 3. **Elemental maps derived from Laser Ablation Time-of-Flight Inductively-Coupled Mass Spectrometry (LA-TOF-ICP-MS) of *Varanus komodoensis* tooth serrations.**

Page 6 Supplementary Figure 4. **Serration and tooth tip colouration in museum specimens of *Varanus*.**

Page 7 Supplementary Figure 5. **Comparisons of tooth crown colouration in other varanid and non-varanid squamates.**

Page 8 Supplementary Figure 6. **Comparisons of tooth crown colouration, iron and zinc sequestration along cutting edges in extant crocodylian teeth.**

Page 9 Supplementary Figure 7. **Comparisons of elemental compositions of extant and fossil crocodylian teeth.**

Page 11 Supplementary Figure 8. **Step-height correction of elemental maps for *Alligator mississippiensis* tooth.**

Page 12 Supplementary Figure 9. **Comparisons of Iron X-ray Absorption Near Edge Structure (Fe-XANES) spectra for the iron layers in extant beaver, crocodile, and Komodo dragon.**

Page 13 Supplementary Figure 10. **Laser-Stimulated Fluorescence (LSF) imaging of cutting edges in selection of fossil theropod teeth from the NHMUK collections.**

Page 14 Supplementary Figure 11. **LA-ICP-MS elemental maps for two tyrannosaurid teeth.**

Page 15 Supplementary Figure 12. **Additional synchrotron-based X-Ray MicroFluorescence (S-µXRF) elemental maps for two tyrannosaurid teeth.**

Page 16 Supplementary Figure 13. **LA-ICP-MS elemental maps for a dromaeosaurid dinosaur tooth (UALVP 61165).**

Page 17 Supplementary Figure 14. **Representative XRF spectra for extant reptile and tyrannosaurid teeth examined in this study.**

Page 18 Supplementary Figure 15. **Scanning Electron Microscope (SEM) imaging of enamel microstructure across tyrannosaurid tooth crowns.**

Page 19 Supplementary Figure 16. **Scanning Electron Microscope (SEM) imaging of enamel microstructure in three dromaeosaurid teeth.**

Page 20 Supplementary Figure 17. **Histological comparisons of wavy enamel along tyrannosaurid serrations and hadrosaurid teeth.**

Page 21 Supplementary Figure 18**. Schematic representation of the machine learning based pipeline used to cluster orientation data by similarity to facilitate parameter extraction and 2D fitting of the 002-diffraction peak(s).**

Page 22 Supplementary Figure 19. **Synchrotron-based X-Ray Micro-diffraction (S-µXRD) maps of two serrations in longitudinal section (UALVP 53472).**

Page 23 Supplementary Figure 20. **Synchrotron-based X-Ray Micro-diffraction (S-µXRD) map of a horizontal section through a serration and the surrounding enamel (UALVP 60554) with statistical comparisons of Full-Width Half Maxima (FWHM) of enamel on- and off-serration.**

Page 24 Supplementary Figure 21. **Nanoindentation analysis of *Varanus komodoensis* J94036-2.**

Page 25 Supplementary Figure 22. **Comparisons of hardness and reduced elastic moduli in two *Alligator mississippiensis* teeth, with a focus on the iron-enriched enamel layers.**

Page 27 Supplementary Figure 23. **Nanoindentation analysis of a tyrannosaurid tooth (UALVP 60555).**

Page 28 Supplementary Figure 24. **Comparisons of indentation hardness and reduced elastic moduli of extant *Varanus komodoensis*.**

Page 29 Supplementary Figure 25. **Comparisons of tooth wear in *Varanus komodoensis* and tyrannosaurid teeth.**

Page 30 Supplementary Figure 26. **Scanning Electron Microscope (SEM) imaging of acid-etched serrations in *Varanus komodoensis*, showing acid-resistance of the outer iron-rich coating.**

Page 31 Supplementary Table 1. **Survey of tooth pigmentation in reptiles.**

Page 35 Supplementary Table 2. **Synchrotron experiment parameters.**

Page 38 Supplementary Table 3. **Raw data and t-tests comparing Full Width Half Maxima (FWHM) between groups of pixels along Synchrotron X-Ray Microdiffraction map of a cross-section of a tyrannosaurid tooth (UALVP 60554).**

Page 45 Supplementary Table 4. **Raw data and t-tests comparing enamel hardness and elastic modulus along unpigmented and pigmented regions via nanoindentation in an *Alligator* tooth.**

Page 48 Supplementary Table 5: **Experimental parameters and data acquisition parameters used for LA-ICP-MS imaging.**

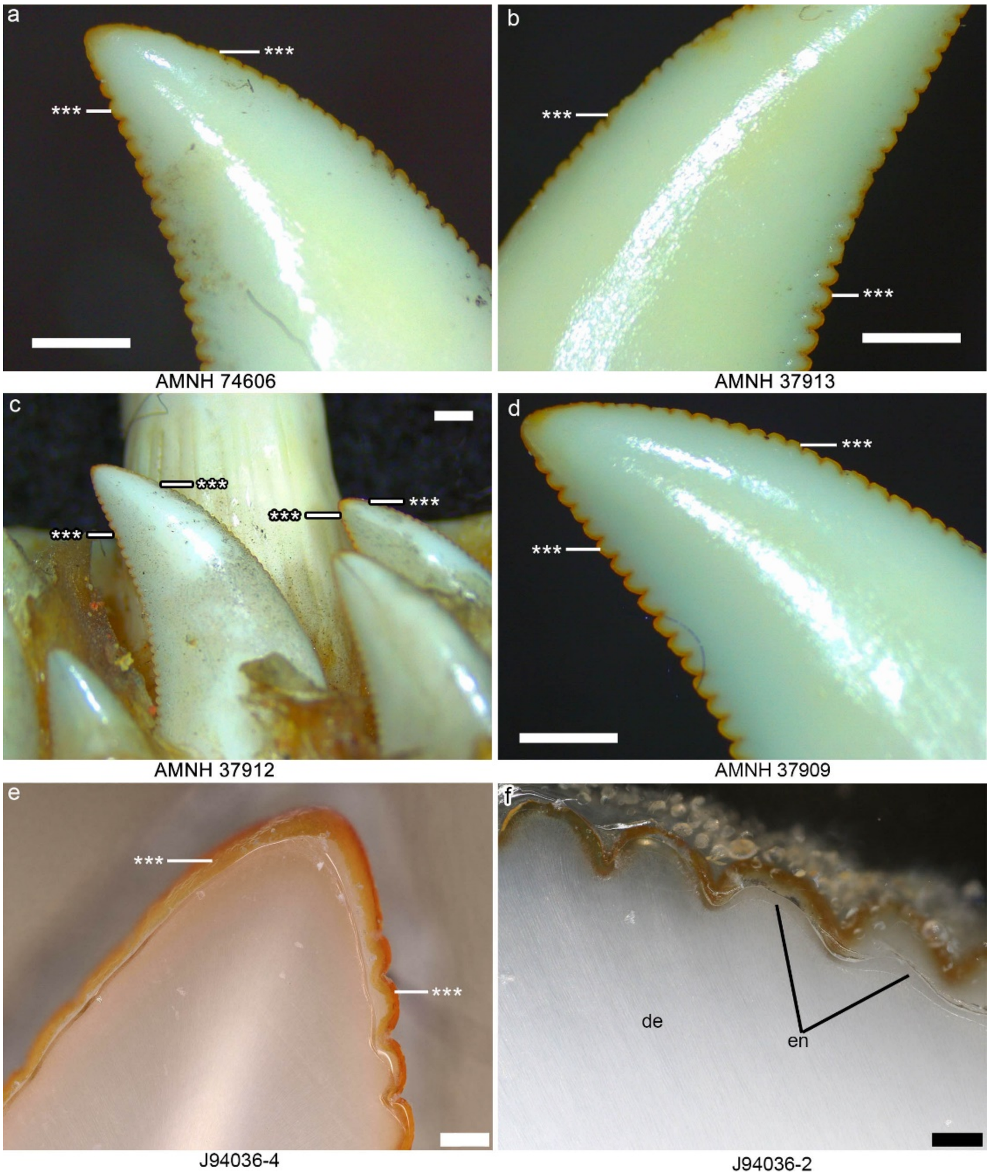

Supplementary Figure 1. **Pigmented cutting edges and tooth tips in museum specimens of *Varanus komodoensis*.** **a** Functional tooth (AMNH 74606). **b** Functional tooth (AMNH 37913). **c** Replacement teeth, from below the gumline and unworn (AMNH 37912). **d** Functional tooth (AMNH 37909). **e** Polished thick section of a functional tooth; the pigmented region is still embedded in a thin layer of resin (J94036-4). **f** Polished thick section along the mesial serrations of a functional tooth; the clear enamel is exposed on the surface of the polished block and the pigmented regions are still embedded in resin (J94036-2). Asterisks indicate orange pigmented regions. Abbreviations: AMNH American Museum of Natural History (New York, New York, USA), de dentine, en enamel. Scale bars in a-d are 1mm, e-f are 0.1mm.

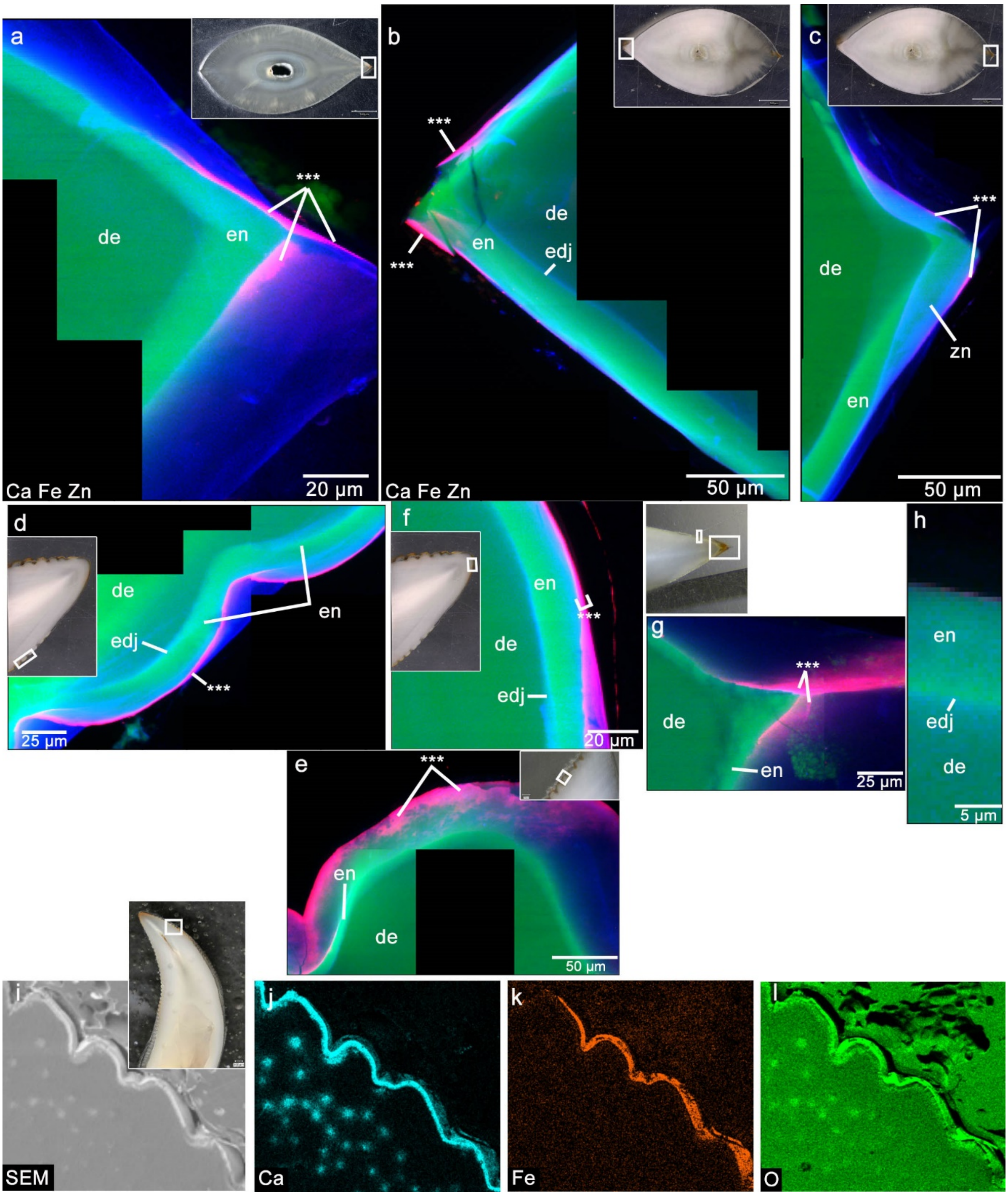

Supplementary Figure 2. **Additional synchrotron-based X-Ray MicroFluorescence (S-μXRF) and Scanning Electron Energy-Dispersive x-ray Spectroscopy (SEM-EDS) elemental maps for *Varanus komodoensis* teeth. a** S-μXRF map (0.5 μm resolution) of iron (red), calcium (green), and zinc (blue) in a horizontal thick section taken through an unerupted tooth crown (MoLS X-263). Map shows iron and zinc sequestration along the outer enamel of a distal serration. **b** S-μXRF map (0.5 μm resolution) of iron (red), calcium (green), and zinc (blue) in a horizontal thick section taken through the same tooth crown. Map shows iron and zinc sequestration along the outer enamel of another distal serration of MoLS X-263 (same as main text Fig. 2g). **c** S-μXRF map (0.5 μm resolution) of iron (red), calcium (green), and zinc (blue) in the same horizontal thick section taken through MoLS X-263 as in b. Map shows iron

and zinc sequestration along the outer enamel of a mesial serration of MoLS X-263 (same as main text Fig. 2i). **d** S-μXRF map (0.5 μm resolution) of iron (red), calcium (green), and zinc (blue) in a longitudinal thick section taken parallel to the serrations. Map shows iron and zinc sequestration along the outer enamel of mesial serrations of X-263. **e** S-μXRF map (0.5 μm resolution) of iron (red), calcium (green), and zinc (blue) in a longitudinal thick section through an erupted, functional tooth crown (J94036-1). Map shows iron and zinc sequestration along the outer enamel of a distal serration. **g** S-μXRF map of a distal serration of J94036-5 (0.5 μm resolution). **h** S-μXRF map through the enamel and dentine off-serration (0.5 μm resolution). Note the lack of prominent iron signal. **i** Scanning Electron Microscope image of mesial serrations of an erupted, functional tooth (J94036-2). **j** SEM-Energy Dispersive Spectroscopic image of calcium, **k** iron, and **l** oxygen along the mesial serrations of J94036-2. Abbreviations: de dentine, edj enamel-dentine junction, en enamel, zn zinc-enriched region of enamel. Asterisks refer to iron-coated regions.

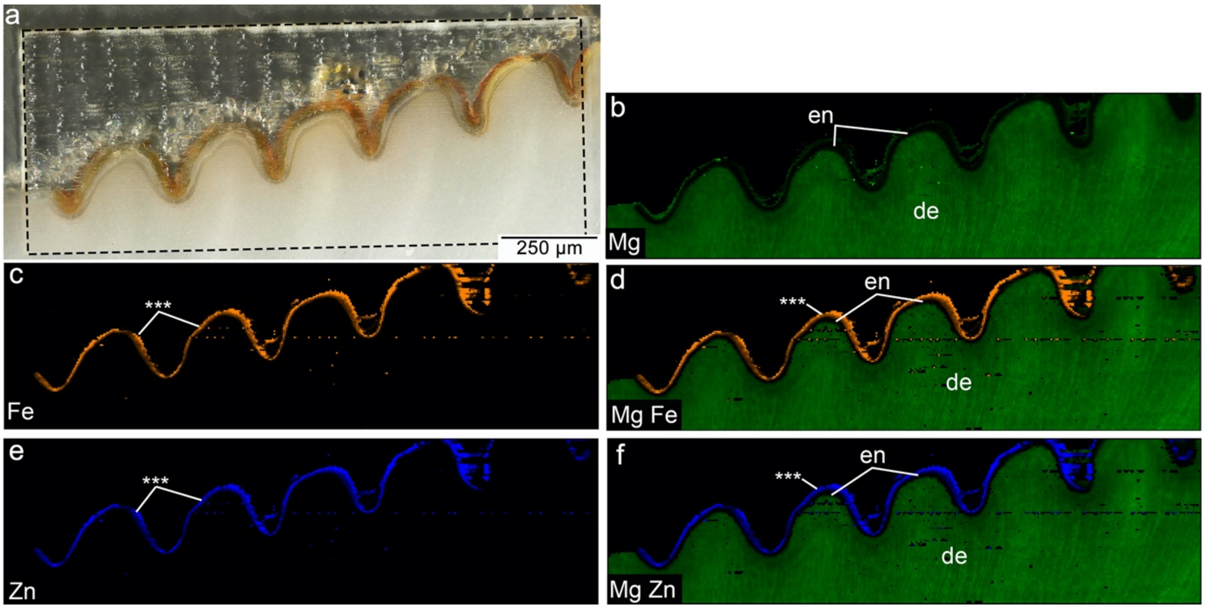

Supplementary Figure 3. **Elemental maps derived from Laser Ablation Time-of-Flight Inductively-Coupled Mass Spectrometry (LA-TOF-ICP-MS) of *Varanus komodoensis* tooth serrations.** Maps were first normalized to the calcium counts to account for artefacts that arose from differential ablation of enamel vs dentine (see Methods and Supplementary Fig. 8 and text therein for explanation). The calcium map is therefore not shown. **a** White light image of ablated region of distal serrations of J94036-1 (dashed lines). **b** Map of magnesium, showing higher counts in the dentine. **c** Iron map showing its restriction to the outer layer of enamel. **d** Map of iron and magnesium. **e** Zinc map, showing its restriction to the outer enamel layer. **f** Map of zinc and magnesium. Asterisks indicate position of pigmented enamel. Abbreviations: de dentine, en enamel.

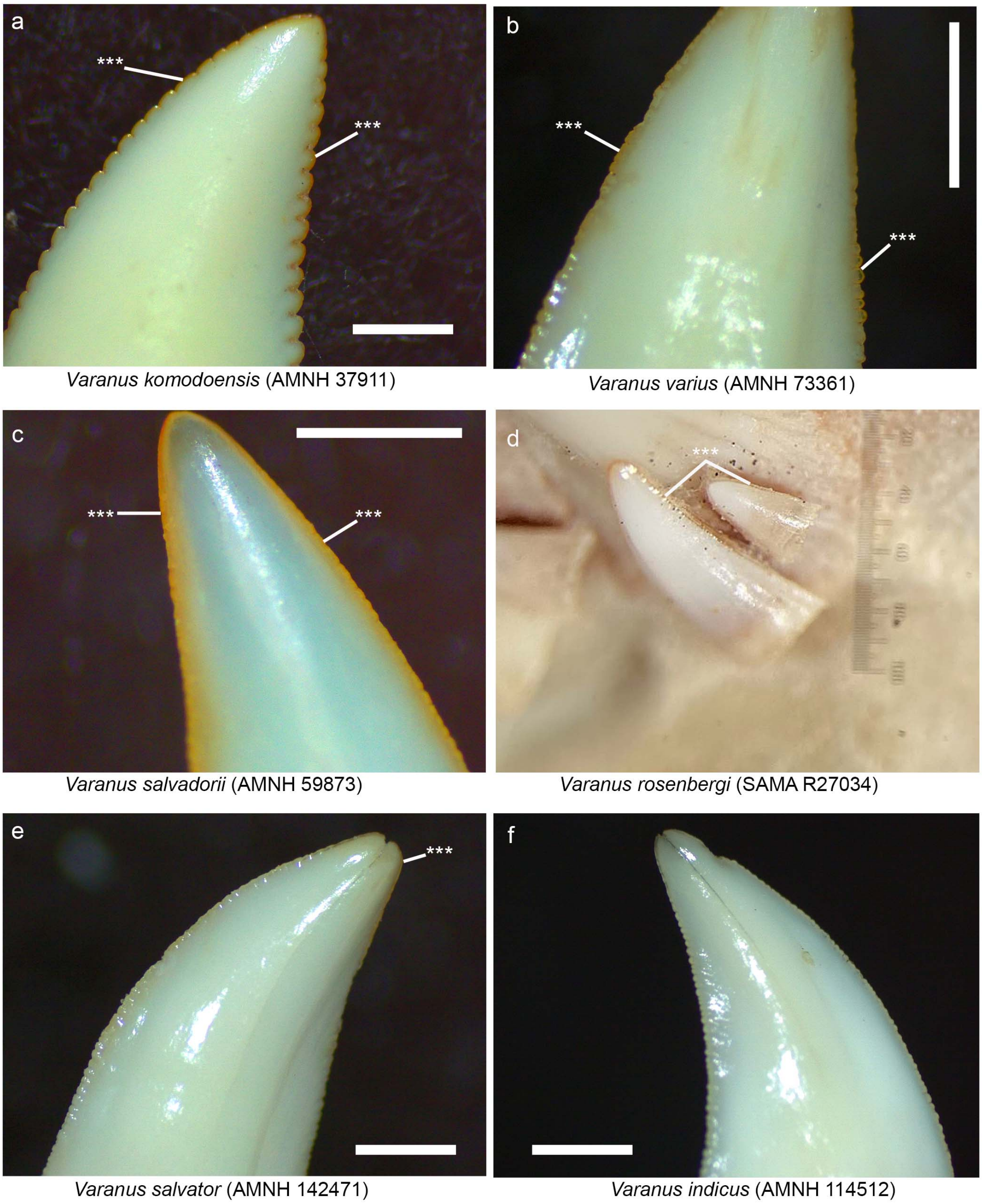

Supplementary Figure 4. **Serration and tooth tip colouration in museum specimens of *Varanus*.** All images taken in lingual or labial views. Asterisks indicate orange pigmentation. All scale bars except the one in d are 1mm. Abbreviations: AMNH American Museum of Natural History, SAMA South Australian Museum.

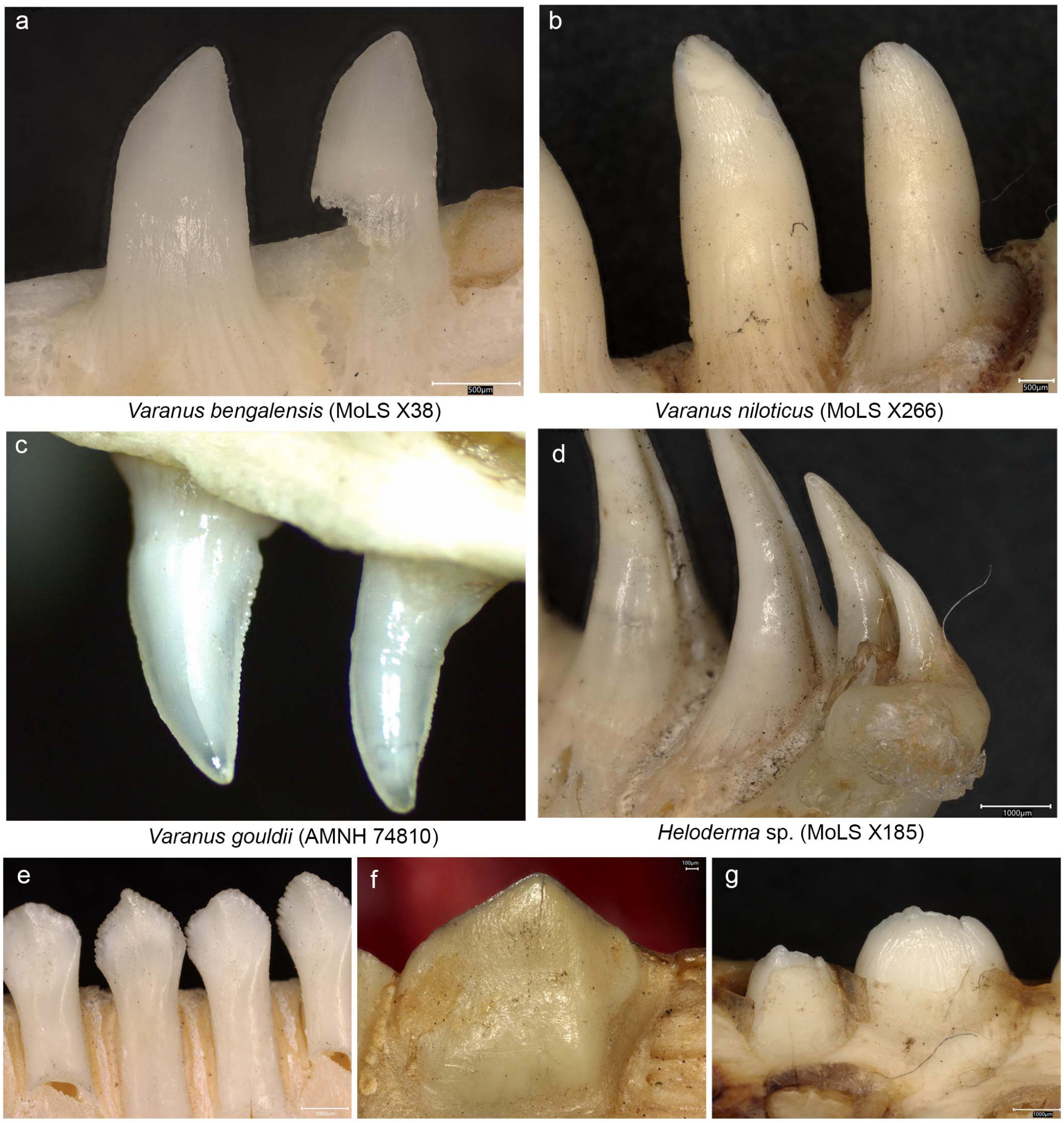

Varanus bengalensis (MoLS X38)

Varanus niloticus (MoLS X266)

Varanus gouldii (AMNH 74810)

Heloderma sp. (MoLS X185)

Iguanidae indet. (MoLS X48)

Agamidae indet. (MoLS X68)

Tupinambis sp. (MoLS X259)

Supplementary Figure 5. **Comparisons of tooth crown colouration in other varanid and non-varanid squamates.** All images taken in lingual views. Abbreviations: AMNH American Museum of Natural History, MoLS, Museum of Life Sciences (King's College London).

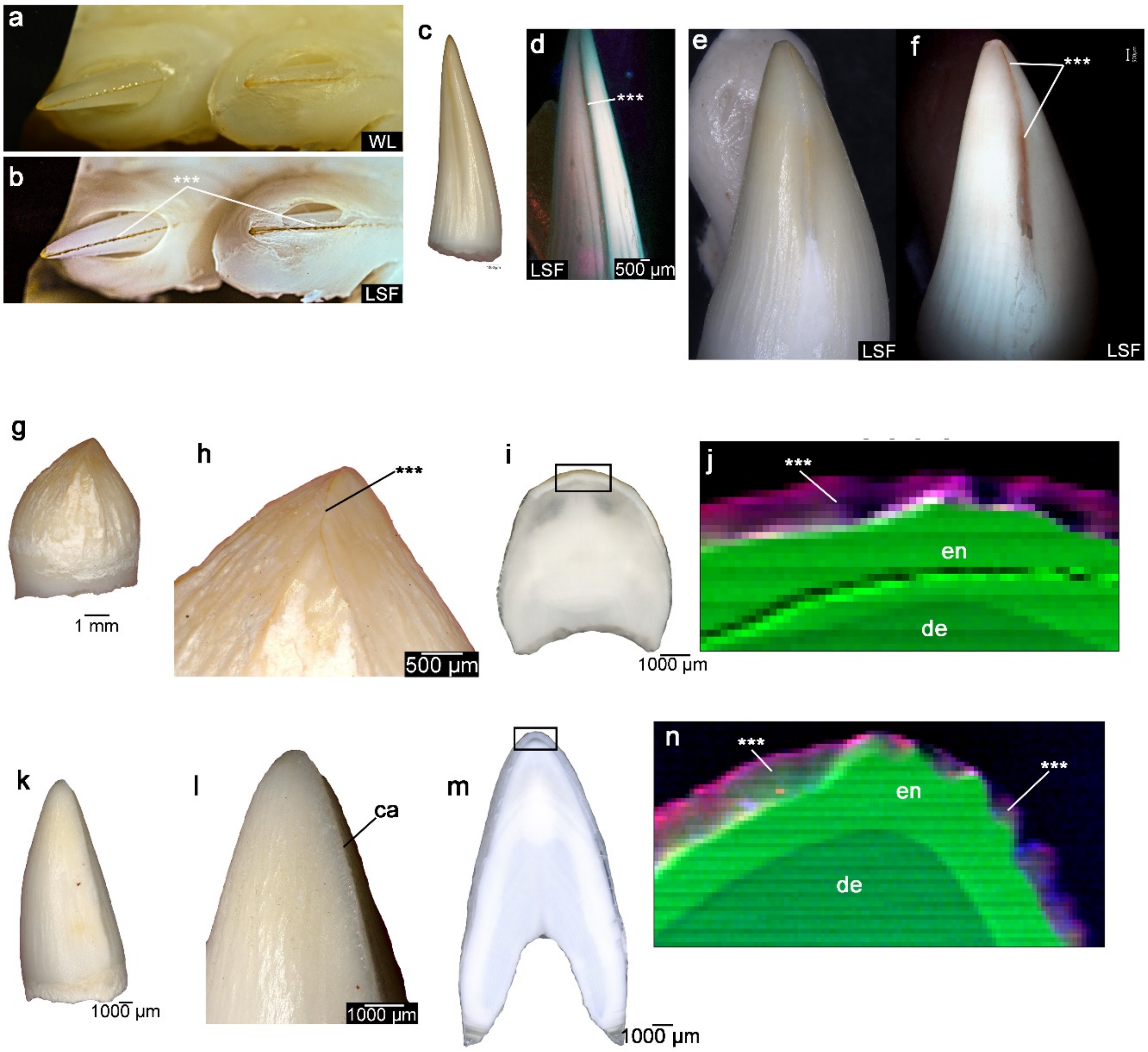

Supplementary Figure 6. **Comparisons of tooth crown colouration, iron and zinc sequestration along cutting edges in extant crocodylian teeth.** **a** White light (WL) and **b** Laser Stimulated Fluorescence images of two teeth from a *Varanus komodoensis* (Zoological Society of London) showing differential fluorescence of the serrations (asterisks) for comparisons with crocodylian samples. **c** Anterior tooth of *Tomistoma schlegelii* in distal view. **d** LSF image of distal carina, showing different fluorescence patterns between carina (asterisks) and the rest of the tooth crown, similar to *V. komodoensis*. **e** Posterior tooth of *T. schlegelii* under white light (WL) and **f** Laser Stimulated Fluorescence (LSF) showing differential fluorescence of carina (asterisks). **g** Mesial view of a posterior tooth crown of *Osteolaemus tetraspis*. **h** Closeup of tooth tip showing pigmented cutting edges. **i** Longitudinal section taken through mesial and distal cutting edges of tooth in g. **j** Synchrotron X-Ray Microfluorescence (S-µXRF) map of iron (red), calcium (green), and zinc (blue) taken from tip of tooth section in h. Iron and zinc are restricted to the outermost enamel layers along the tooth tip and cutting edges. **k** Mesial view of a tooth crown of *Crocodylus porosus*. **l** Closeup of tooth tip in j, showing a lack of obvious pigmentation along the carina under white light. **m** Longitudinal section taken through the mesial and distal carinae for S-µXRF analysis. **n** S-µXRF map of iron (red), calcium (green), and zinc (blue), showing iron and zinc sequestration along the tip and cutting edge enamel in the same tooth as in l. Abbreviations: ca carina, de dentine, en enamel

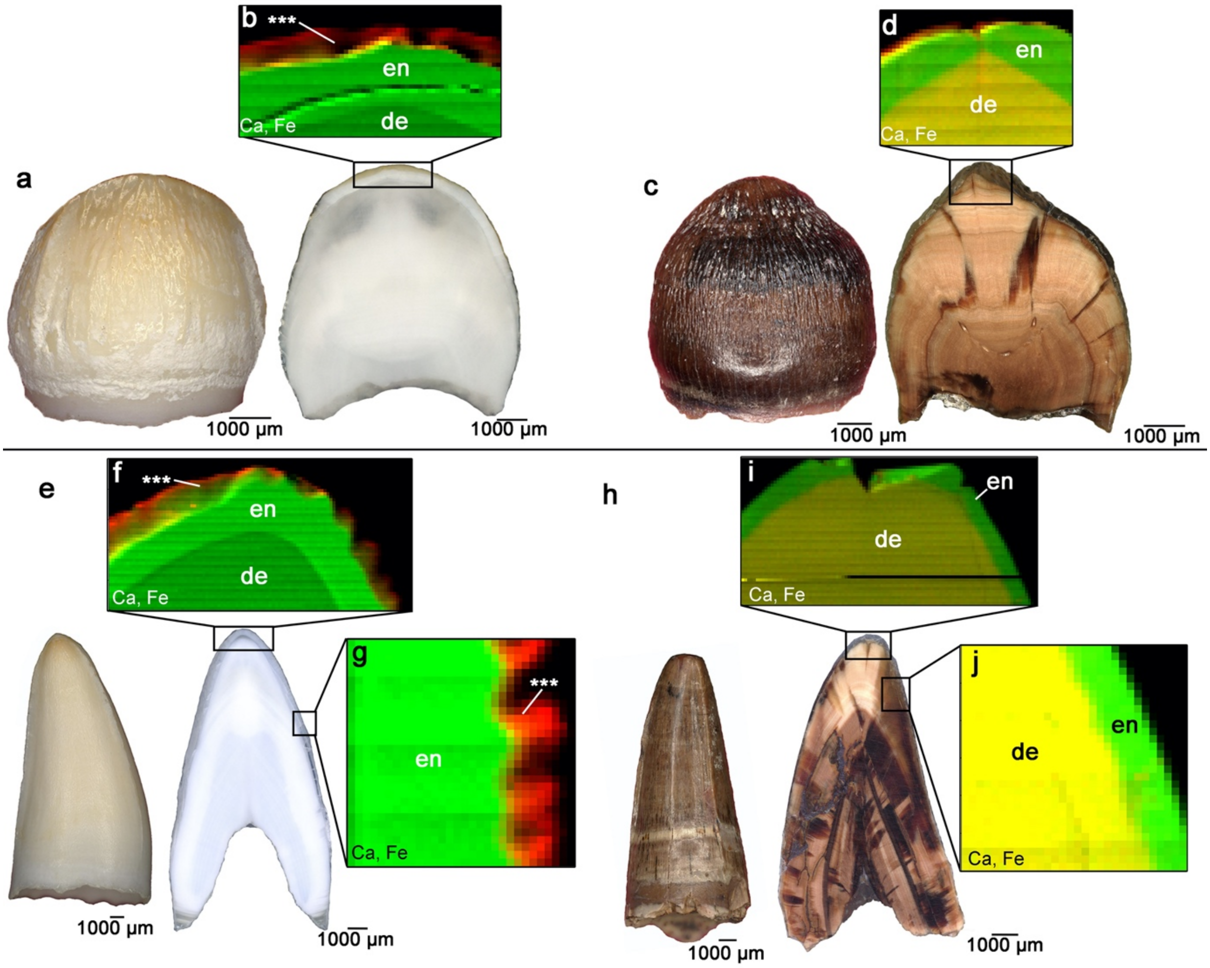

Supplementary Figure 7. **Comparisons of elemental compositions of extant and fossil crocodylian teeth. a** Posterior tooth of *Osteolaemus tetraspis* before (left) and after sectioning along mesiodistal axis (right). **b** S-µXRF map of iron (red) and calcium (green) along the tooth tip. Iron is located only in the outermost enamel layers. **c** Posterior tooth of a fossil crocodylian from Dinosaur Provincial Park (UALVP 60546) with similar morphology to *O. tetraspis* before (left) and after sectioning along mesiodistal axis (right). **d** S-µXRF map of iron (red) and calcium (green) showing the abundance of iron within the dentine and enamel. **e** Anterior tooth of *Crocodylus porosus* before (left) and after sectioning along the mesiodistal axis (right). **f** S-µXRF map of iron (red) and calcium (green) along the tooth tip and **g** along the carina. **h** Anterior tooth of a fossil crocodylian from Dinosaur Provincial Park (UALVP 60550) with similar morphology to *C. porosus* before (left) and after sectioning along mesiodistal axis (right). **i** S-µXRF map of iron (red) and calcium (green) along the tooth tip showing the abundance of iron within the dentine and enamel. **j** S-µXRF map of iron (red) and calcium (green) along a carina showing the abundance of iron within the dentine and enamel. Abbreviations: de dentine, en enamel. Asterisks indicate positions of iron-enriched enamel.

Laser Ablation Inductively-Coupled Plasma Mass Spectrometry (LA-ICP-MS) data processing

Elemental maps generated from the LA-ICP-MS experiments were sensitive to the mechanical properties of each tissue. For example, for a given transect of ablation along the extant *Alligator*

tooth sample, the laser removed more dentine (which is softer) from the tooth than enamel (which is significantly harder). Consequently, element counts were underrepresented in the enamel relative to the dentine. This bias was especially evident in raw elemental maps for calcium, where the results initially indicated higher calcium counts in the dentine than in the enamel, which was opposite to all XRF and SEM-EDS data. To account for this artifact, we applied a correction factor on the LA-ICP-MS count data for iron, calcium, and zinc.

To implement a correction to the raw element counts, we first estimated the amount of enamel lost during the ablation process and compared it with the amount dentine (Supplementary Fig. 8d, f). We calculated the depths of ablation along the enamel and dentine by generating profile lines from z-stacked microscope images of the tooth surface using the Keyence digital microscope's 3-D imaging function. We calculated an average step height for dentine in *Alligator* tooth 2 ROI1 of -5.43 µm, whereas we could not reliably detect a step height through the enamel. We therefore had to rescale the elemental maps for iron, calcium, and zinc for this tooth, given that we ablated approximately 5.4 times more dentine than enamel. We did this by manually masking every pixel in the dentine in ImageJ and then dividing this region in each elemental map by the difference in step height (5.433). This resulted in re-scaled maps where the counts between enamel and dentine were comparable with elemental data obtained from the other techniques.

To more accurately depict the relative differences in elemental concentrations, each map needed to be processed differently. For the iron map, the counts for iron along the surface of the enamel were approximately three orders of magnitude higher than for the counts in the rest of the enamel and dentine. We therefore rescaled this map to a log scale (from background to ~3000 counts in the outer enamel). However, zinc and calcium showed much smaller differences along the outer and inner enamel, as well as the dentine. We presented the zinc map from 0 (background) to the 95th percentile to eliminate outliers and produce a more realistic distribution of zinc through the tooth. Calcium also showed much smaller count differences between enamel and dentine and is therefore also presented on a linear scale from 0 to the 95th percentile to eliminate outliers. The corrected maps here and in the main text are therefore relative representations of the concentrations of iron, calcium, and zinc, and therefore do not include count scale bars for this reason. We also could not detect any difference in step height between ablated enamel and ablated dentine in the fossil tyrannosaurid teeth and therefore present the LA-ICP-MS elemental maps for these teeth without any correction (Supplementary Figs. 11, 13).

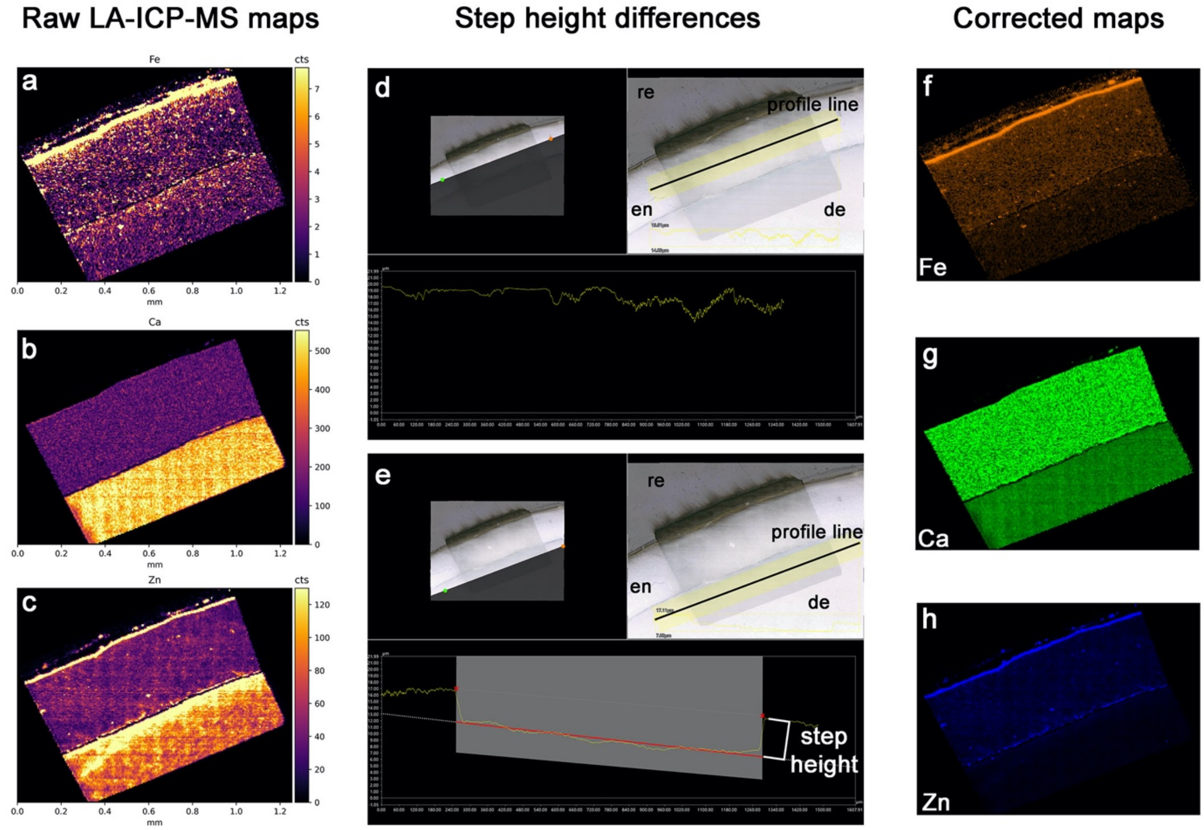

Supplementary Figure 8. **Step-height correction of elemental maps for *Alligator mississippiensis* tooth.** **a** Raw iron map from LA-ICP-MS. **b** raw calcium map. Note the higher counts of calcium in the dentine (lower region) compared with the enamel. This is in direct opposition to all other calcium maps generated from XRF and EDS analyses. **c** Raw zinc map. Note higher zinc counts in the dentine compared with the enamel. **d** Profile line drawn through the ablated region of enamel in the tooth sample using the profile function through a z-stacked image of the sample using the Keyence VHX digital microscope. No consistent step height could be detected, suggesting negligible ablation of the enamel surface. **e** Profile line drawn through the dentine. We measured a step height of 5.43µm between ablated and unablated regions of the dentine. This was used as a correction factor for the derived elemental maps. **h** corrected map for iron, **i** calcium, and **j** zinc. Note reversal of counts for zinc and calcium between enamel and dentine in the corrected maps. Abbreviations: de dentine, en enamel, re resin.

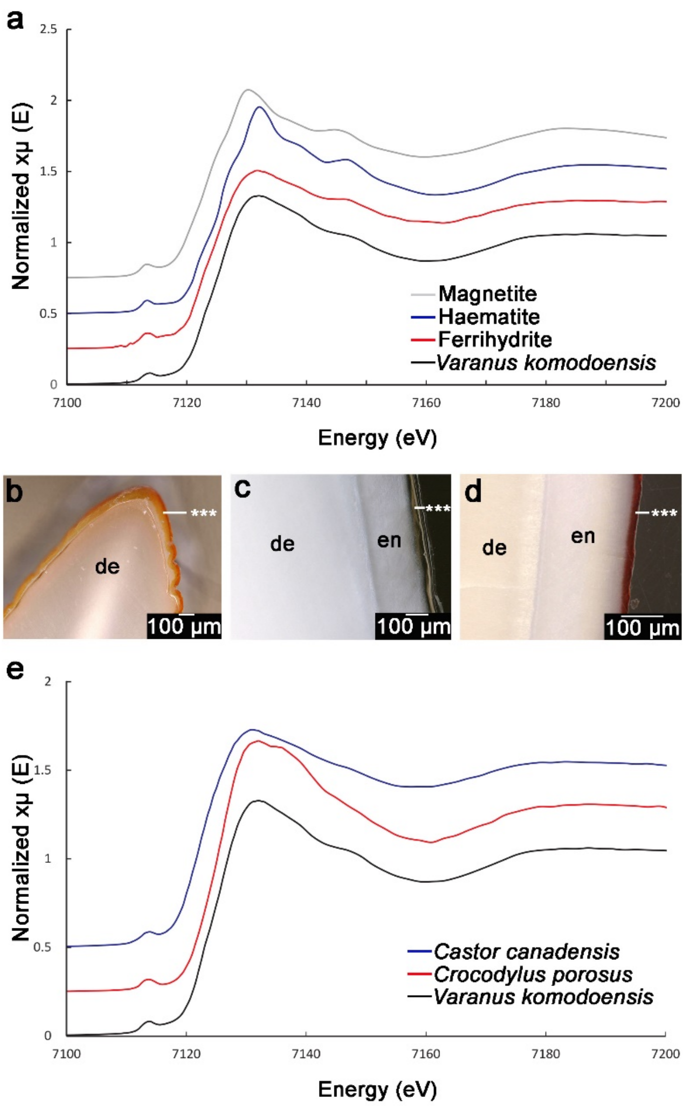

Supplementary Figure 9. **Comparisons of Iron X-ray Absorption Near Edge Structure (Fe-XANES) spectra for the iron layers in extant beaver, crocodile, and Komodo dragon. a** Comparisons of XANES spectra for magnetite, haematite, and ferrihydrite standards with the spectra derived from the iron coatings in a *V. komodoensis* tooth. The *V. komodoensis* spectra most closely resembled that of ferrihydrite. **b** Closeup of iron coatings in a polished thick section of a *V. komodoensis* tooth (J94036-4). **c** Closeup of iron layer within the outer enamel of a *A. mississippiensis* tooth ("Tooth 2"). **d** Closeup of iron layer within the outer enamel of a *Castor canadensis* tooth (UALVP 56017-3). **e** Comparisons of Fe-XANES spectra of the iron layers in *V. komodoensis*, *C. porosus*, and *C. canadensis*. Though consistent with ferrihydrite, the iron layers in *V. komodoensis* differ from those of the iron layers in the other two species. Abbreviations: de dentine, en enamel. Asterisks indicate positions of pigmented enamel layers.

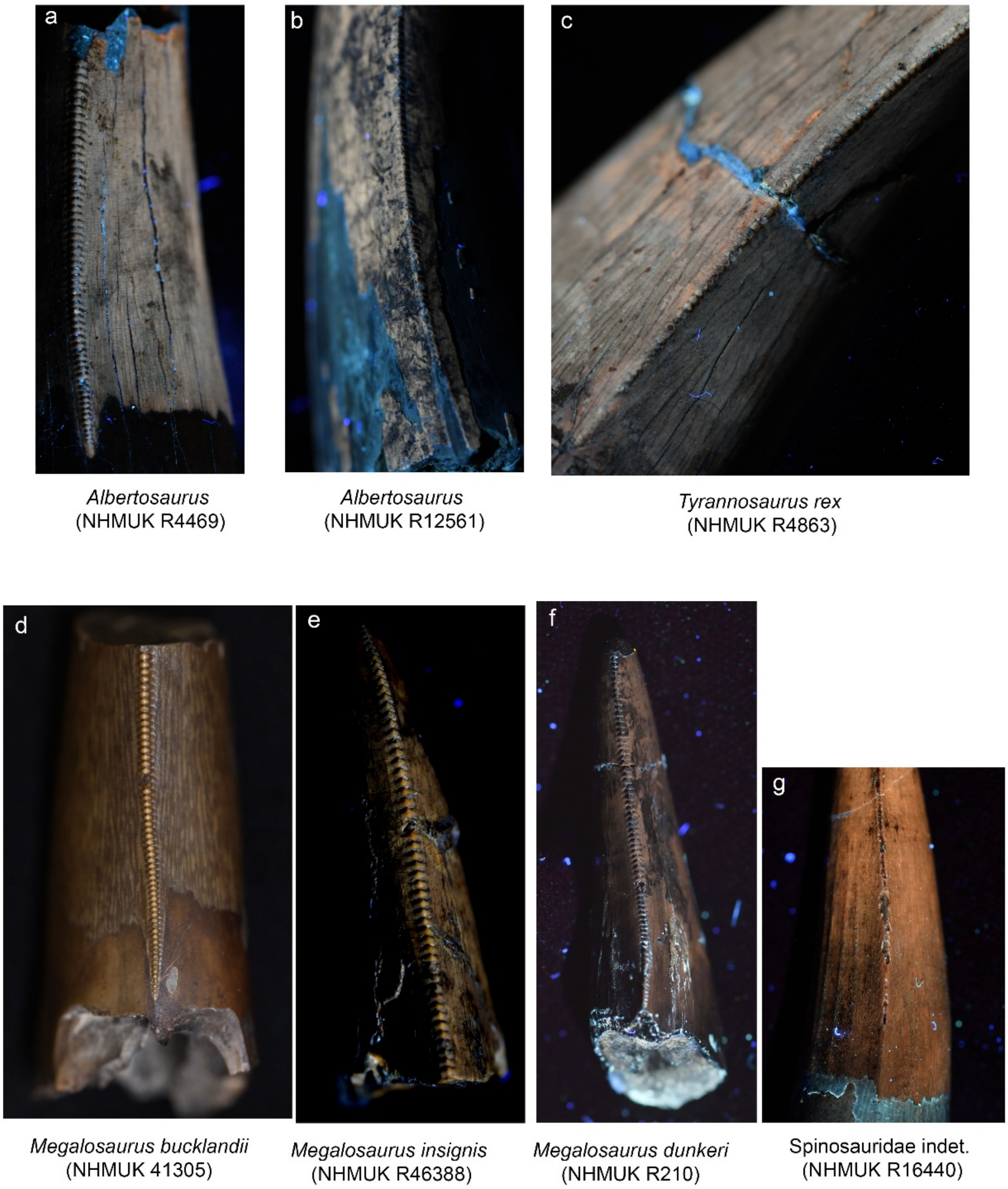

Supplementary Figure 10. **Laser-Stimulated Fluorescence (LSF) imaging of cutting edges in selection of fossil theropod teeth from the NHMUK collections.** Note that none of the samples show differential colouration along the cutting edges (worn edges appear darker due to the exposure of underlying dentine). **a** Distal serrations of a tooth of the tyrannosaurid *Albertosaurus* showing no differences in fluorescence pattern between serrations and the rest the crown. **b** Distal serrations of another *Albertosaurus* tooth showing similar fluorescence patterns between serrations and rest of crown. Blue regions are areas covered in adhesives. **c** Distal serrations of a tooth of *Tyrannosaurus rex* showing no differential fluorescence patterns along the crown. Blue colour is the result of the fluorescence of adhesives. **d** Distal serrations of a partial crown of the megalosaurid *Megalosaurus bucklandii*. Serrations show no differential fluorescence compared with the remainder of the crown. **e** Distal serrations along a tooth of the theropod "*Megalosaurus*" *insignis* showing no difference between the serrations and remainder of the crown. **f** Distal serrations of a tooth of "*Megalosaurus*" *dunkeri* showing

no difference between serrations and remainder of crown. **g** Distal carina of a spinosaurid tooth showing no difference in fluorescence between cutting edge and remainder of crown. Dark patches along the carina result from breakage of the enamel and exposure of the underlying dentine.

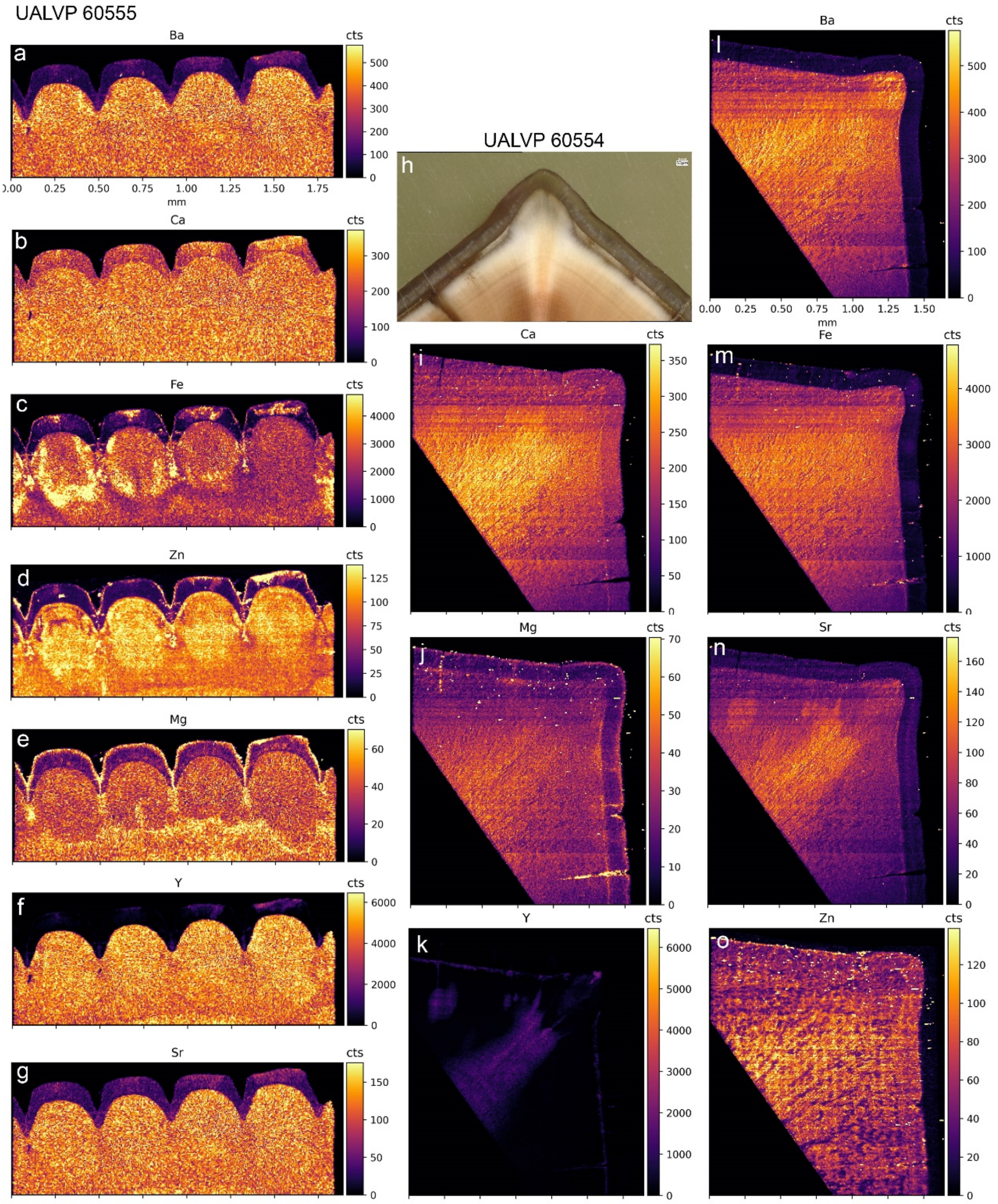

Supplementary Figure 11. **LA-ICP-MS elemental maps for two tyrannosaurid teeth. a** Longitudinal section through distal serrations of UALVP 60555 with elemental map for barium, **b** Calcium, **c** Iron, **d** Zinc, **e** Magnesium, **f** Yttrium, **g** Strontium. **h** White light image of polished thick section through a distal serration of UALVP 60554. **i** elemental map of calcium, **j** Magnesium, **k** Yttrium, **l** Barium, **m** Iron, **n** Strontium, **o** Zinc. None of these

elemental distributions match those seen from elemental analyses of extant *Varanus komodoensis* or crocodylian teeth.

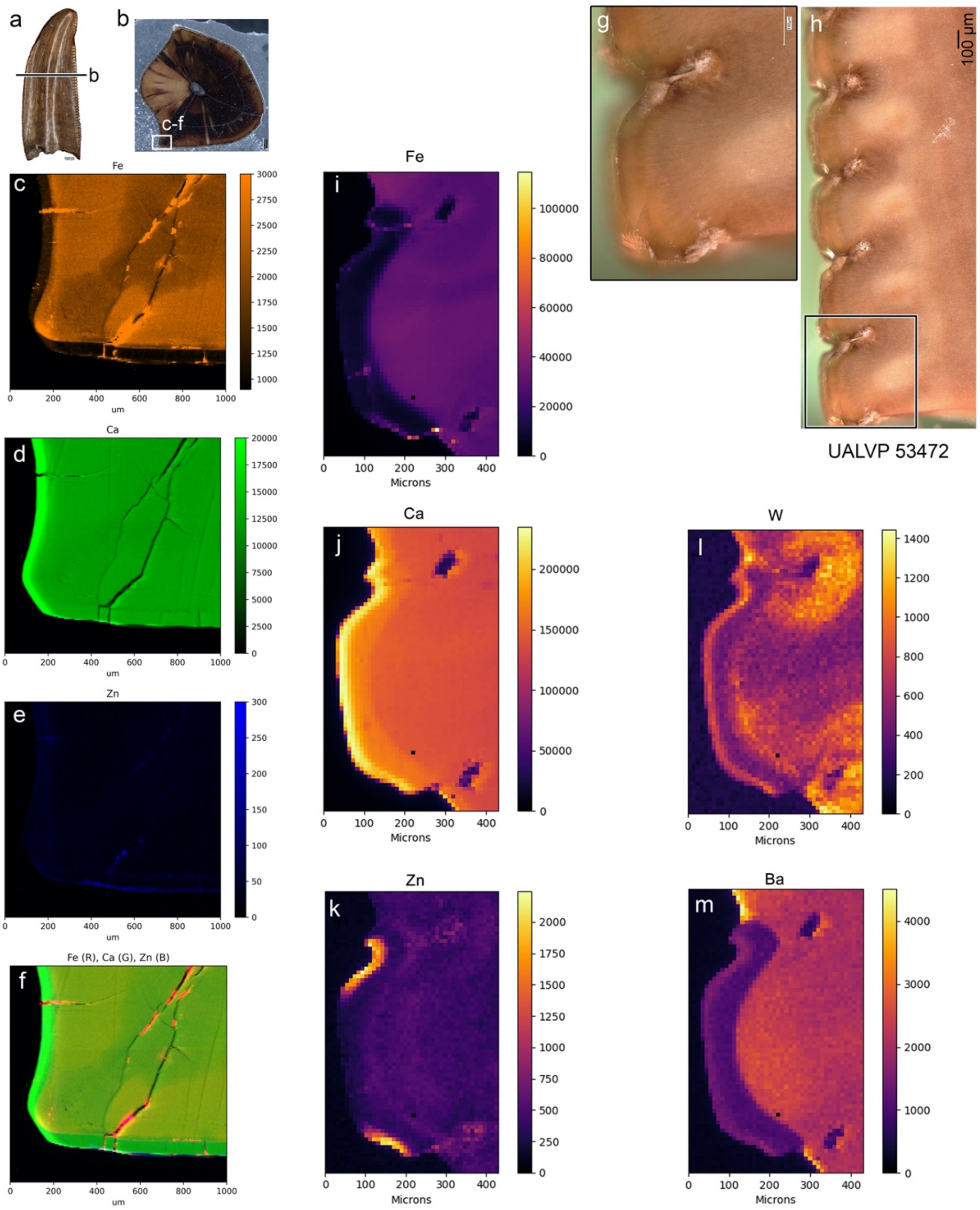

Supplementary Figure 12. **Additional synchrotron-based X-Ray MicroFluorescence (S-µXRF) elemental maps for two tyrannosaurid teeth. a** Distal view of a tyrannosaurid premaxillary tooth (UALVP 60553) used for S-µXRF analyses. **b** Overview image of horizontal section taken through UALVP 60553, showing position of S-µXRF elemental maps in c-f. **c** S-µXRF map of horizontal section through a premaxillary tooth serration, showing distribution of iron, **d** Calcium, **e** Zinc, and **f** Composite of all three elements. Note the lack of iron and zinc sequestration along the serration enamel towards the bottom left of the image. Instead, iron counts are highest in the dentine and along cracks in the tooth, suggesting iron concentrations are primarily driven by fossilization artifacts. **g** Closeup of a longitudinal

section through a mesial serration of another tyrannosaurid tooth (UALVP 53472). **h** Lower magnification image showing position of mapped serration. **i** S-µXRF elemental map for iron, **j** Calcium, **k** Zinc, **l** Tungsten, and **m** Barium. Abbreviations: UALVP University of Alberta Laboratory of Vertebrate Paleontology.

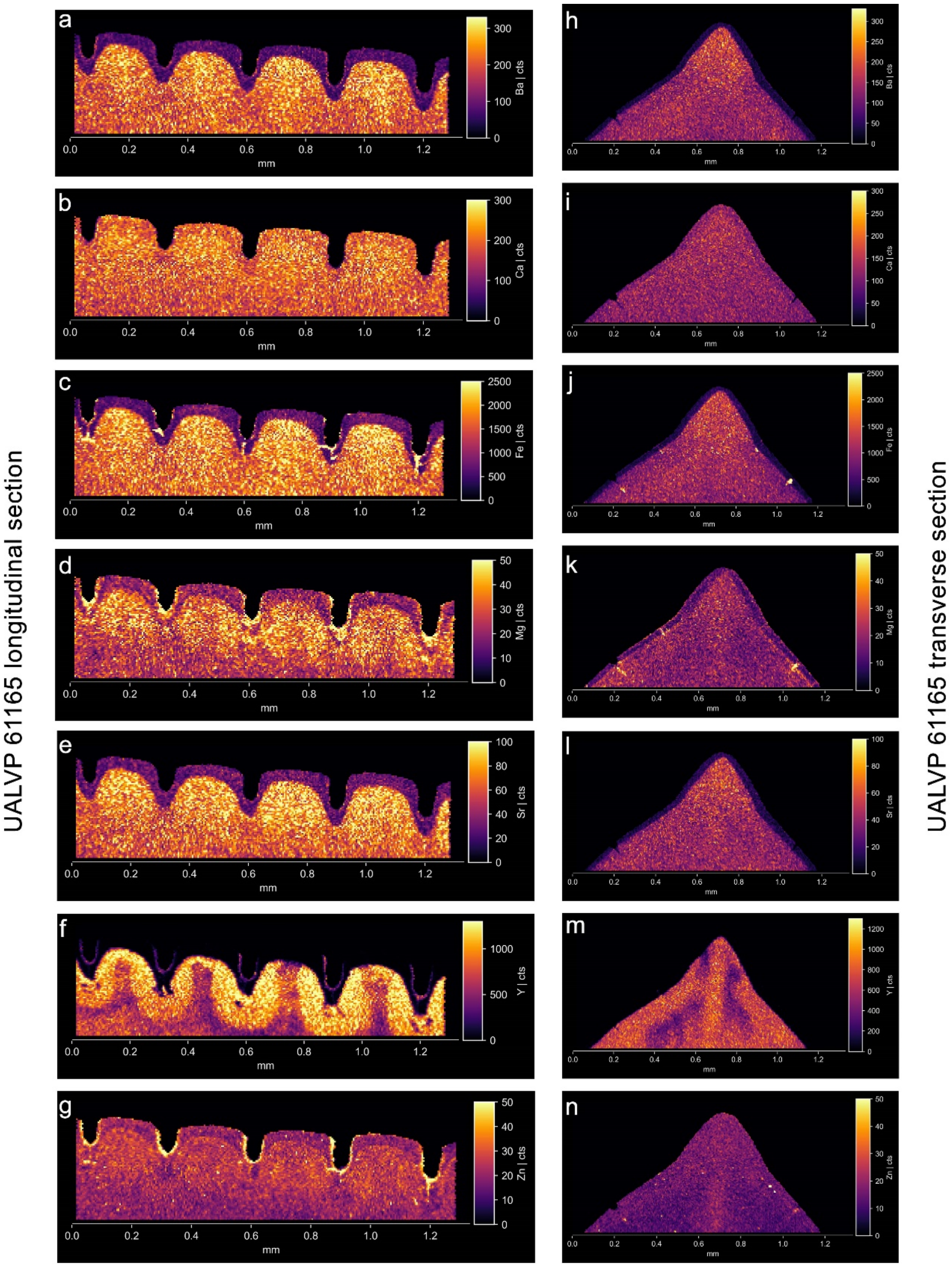

Supplementary Figure 13. **LA-ICP-MS elemental maps for a dromaeosaurid dinosaur tooth (UALVP 61165). a** Longitudinal section through distal serrations of UALVP 61165 with elemental map for barium, **b** Calcium, **c** Iron, **d** Magnesium, **e** Strontium, **f** Yttrium, **g** Zinc. **h**

Transverse section through a distal serration in UALVP 61165 showing the elemental map for barium, **i** Calcium, **j** Iron, **k** Magnesium, **l** Strontium, **m** Yttrium, **n** Zinc. None of these distributions match those of extant *Varanus komodoensis* or crocodylian teeth. Abbreviations: UALVP University of Alberta Laboratory of Vertebrate Paleontology.

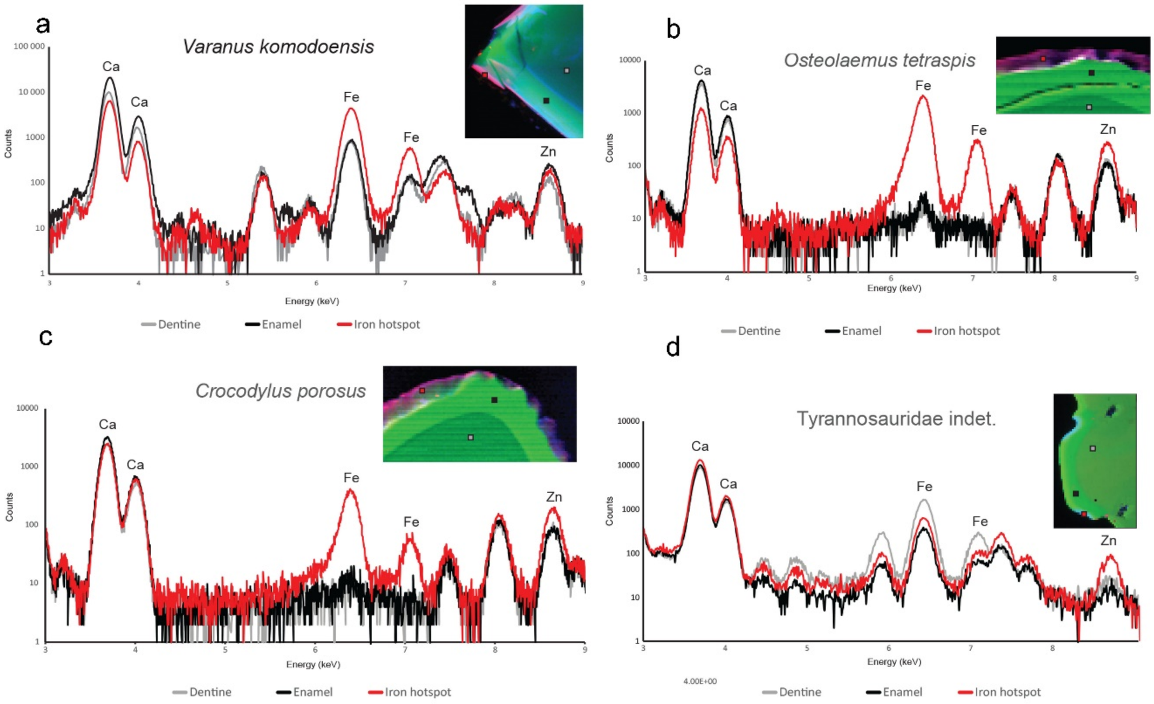

Supplementary Figure 14. **Representative XRF spectra for extant reptile and tyrannosaurid teeth examined in this study. a** XRF spectra taken from the iron-enriched region, enamel, and dentine of a tooth of *Varanus komodoensis* (Beamline ID-21, European Synchrotron Radiation Facility, Grenoble, France). **b** XRF spectra from iron-enriched region, enamel, and dentine of a posterior tooth of *Osteolaemus tetraspsis* (Beamline BM-28, European Synchrotron Radiation Facility, Grenoble, France). **c** XRF spectra from iron-enriched region, enamel, and dentine of an anterior tooth of *Crocodylus porosus* (Beamline BM-28, European Synchrotron Radiation Facility, Grenoble, France). **d** XRF spectra from analogous positions of iron-enriched regions in extant reptiles, enamel, and dentine taken along the serration of a tyrannosaurid tooth (UALVP 53472) (Beamline B-16, Diamond Light Source, Oxfordshire, UK). Note the differences in intensities of iron, calcium, and zinc signals between the three extant reptile teeth and that of the tyrannosaurid. Red boxes in inset elemental map images correspond to regions where "iron hotspot" spectra were taken in each tooth. Grey boxes indicate positions where "dentine" spectra were taken. Black boxes indicate positions where "enamel" spectra were taken.

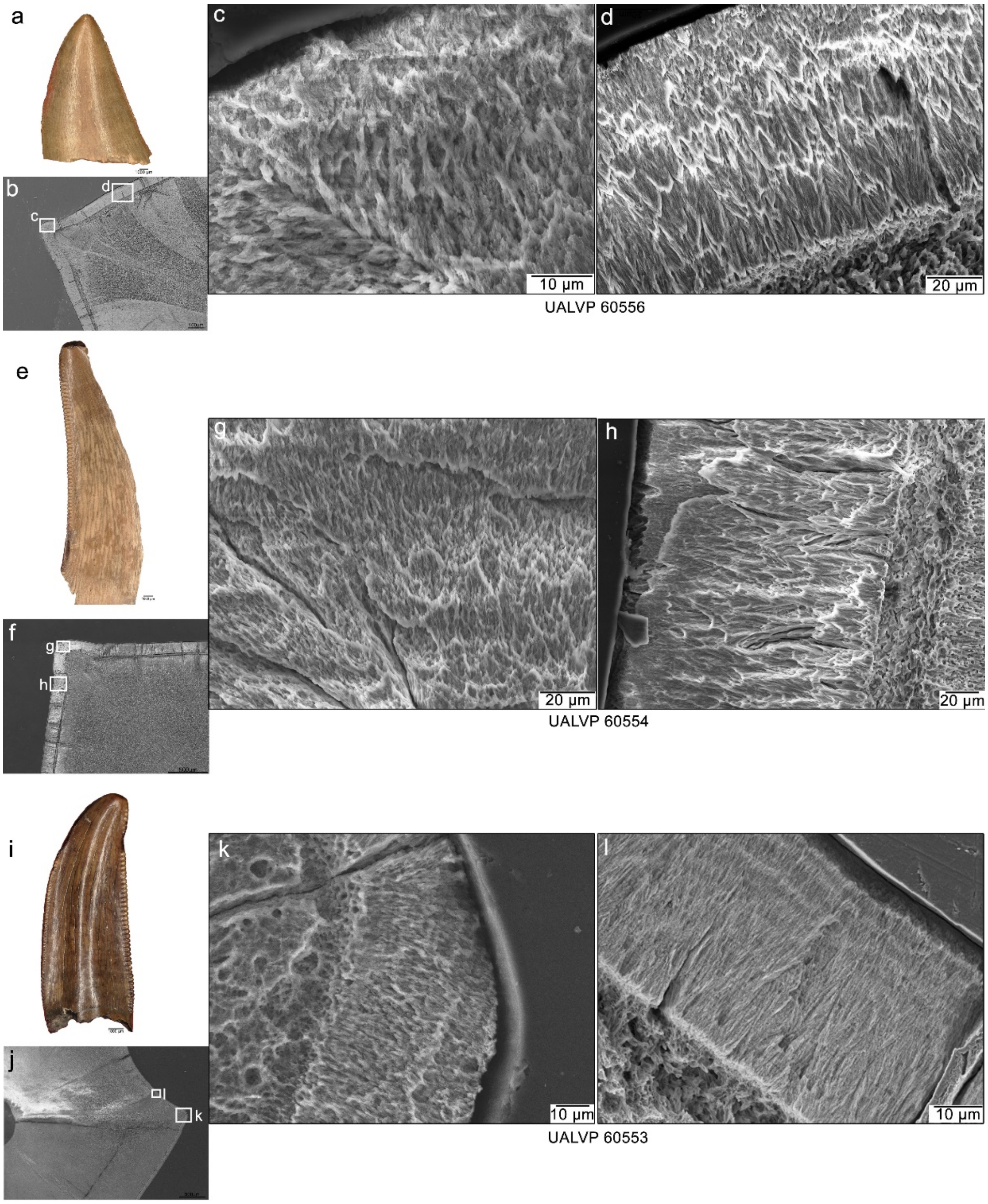

Supplementary Figure 15. **Scanning Electron Microscope (SEM) imaging of enamel microstructure across tyrannosaurid tooth crowns.** **a** Lateral view of the partial tyrannosaurid tooth UALVP 60556. **b** Low-magnification SEM image of horizontal section taken through a distal serration of UALVP 60556. **c** High-magnification SEM image of the wavy enamel along the serration of UALVP 60556. **d** High-magnification SEM image of the columnar enamel found elsewhere on the same tooth crown. **e** Labiolingual view of partial tyrannosaurid tooth (UALVP 60554). **f** Low-magnification SEM image of a horizontal section through one of the distal serrations of UALVP 60554. **g** High-magnification SEM image of the wavy enamel found along the distal serration of UALVP 60554. **h** High-magnification SEM image of columnar enamel along the rest of the same tooth crown. **i** Distal view of a tyrannosaurid premaxillary tooth (UALVP 60553). **j** Low-magnification SEM image of a horizontal section taken through UALVP 60553, showing one of the distal serrations. **k** High-

magnification SEM image of wavy serration enamel in UALVP 60553. **l** High-magnification SEM image of columnar enamel found in other regions of the same tooth crown.

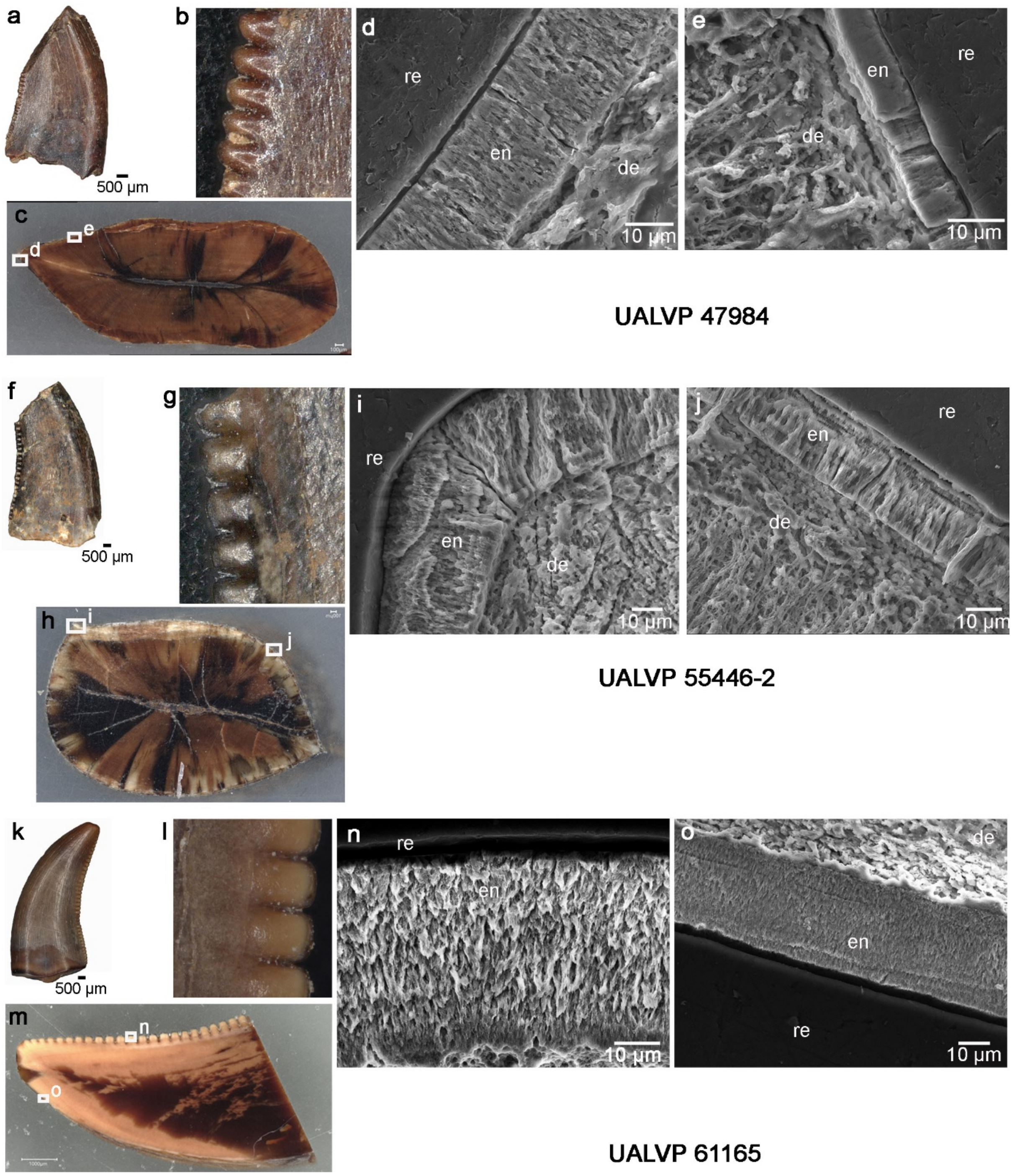

Supplementary Figure 16. **Scanning Electron Microscope (SEM) imaging of enamel microstructure in three dromaeosaurid teeth. a** Partial *Saurornitholestes langstoni* tooth crown sectioned for SEM. **b** Closeup of distal serrations. **c** Wholeview of horizontal section taken through *S. langstoni* tooth used for SEM. **d** SEM image of serration enamel, showing simple parallel crystallite enamel. **e** SEM image of off-serration enamel, showing thinner parallel crystallite enamel. **f** Partial *Dromaeosaurus* sp. tooth crown sectioned for SEM. **g**

Closeup of distal serrations. **h** Wholeview of horizontal section taken through *Dromaeosaurus* sp. tooth for SEM. **i** SEM image of serration enamel showing mostly parallel crystallite enamel, with slight divergences of crystallites along mid-axis of serration. **j** SEM image of off-serration, parallel crystallite enamel. **k** Complete Dromaeosauridae indet. tooth crown sectioned for SEM. **l** Closeup of distal serrations of dromaeosaurid tooth. **m** Wholeview of longitudinal section used for SEM. **n** SEM image of serration enamel, showing microunit and possible wavy enamel (crystallite bundles are not parallel to neighbouring bundles). **o** SEM image of off-serration enamel, showing simpler, parallel crystallites. Abbreviations: en enamel, de dentine, re resin.

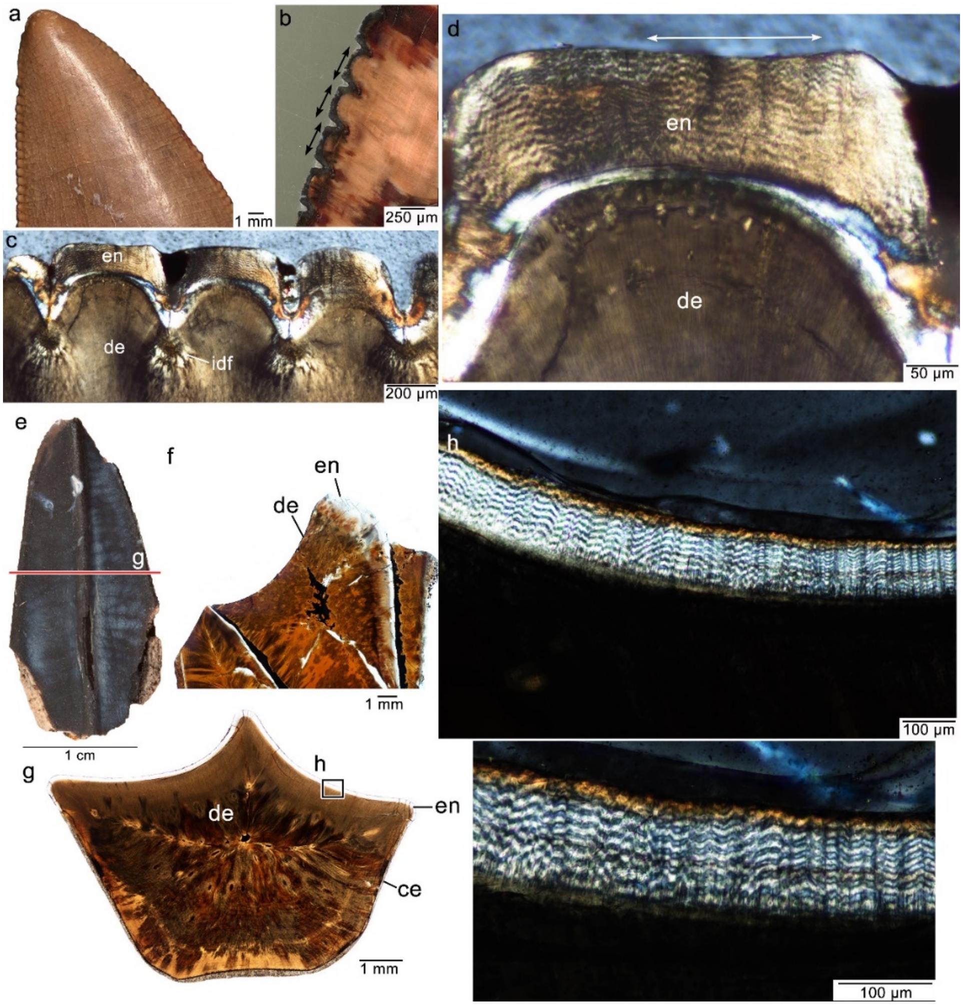

Supplementary Figure 17. **Histological comparisons of wavy enamel along tyrannosaurid serrations and hadrosaurid teeth. a** Lingual view of tyrannosaurid tooth UALVP 60556. **b** Polished thick section through worn mesial serrations of a tyrannosaurid tooth (UALVP 60555). Black arrowheads indicate directions of wear, based on surface striations prior to sectioning (see Suppl. Fig. 15f, g). **c** Longitudinal thin section of serrations in a tyrannosaurid tooth (UALVP 60398) under cross-polarized light. **d** Higher-magnification image of a serration in c, showing wavy enamel effect under cross-polarized light. Arrowheads indicate presumed direction of wear. **e** Isolated hadrosaurid tooth (ROM 58630), showing position of horizontal section in g-i. **f** Longitudinal section through three functional maxillary teeth (image flipped for comparisons) of a hadrosaurid dental battery (ROM 696) showing complexity of the

grinding surface in a hadrosaurid dinosaur. **g** Horizontal thin section through an isolated hadrosaurid tooth (UALVP 55127), showing general histological features and position of higher-magnification images in subsequent panels. **h** Higher magnification image of g under cross-polarized light, showing similar wavy enamel optical effect as that seen in the tyrannosaurid serrations. **i** Higher magnification image of wavy enamel under cross-polarized light. Abbreviations: ce cementum, de dentine, en enamel, idf interdental fold.

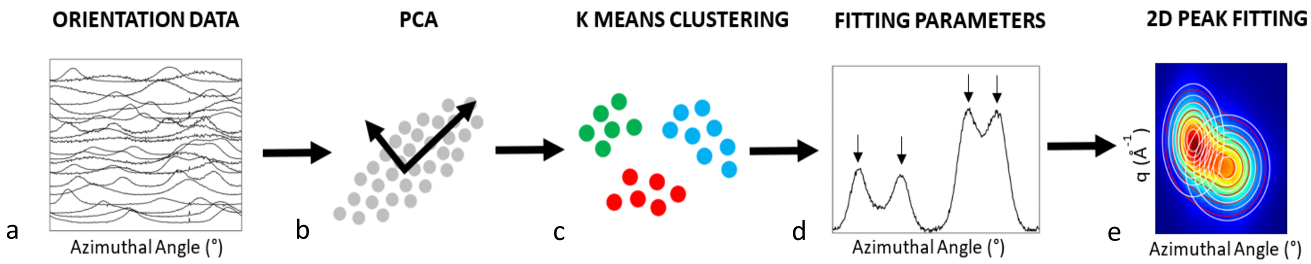

Supplementary Figure 18**. Schematic representation of the machine learning based pipeline used to cluster orientation data by similarity to facilitate parameter extraction and 2D fitting of the 002-diffraction peak(s). a** 1D orientation data, vertically offset for clarity, demonstrates greater variability compared with conventional diffraction data. Data is grouped through the application of **b** principal components analysis and **c** k-means clustering. Fitting parameters, including peak position and width, are extracted from the orientation data **d** and are subsequently used for **e** 2D pseudo-Voigt fitting of diffraction images truncated about the 002 peak(s) to obtain preferred orientation and c axis parameters for constituent crystallite populations.

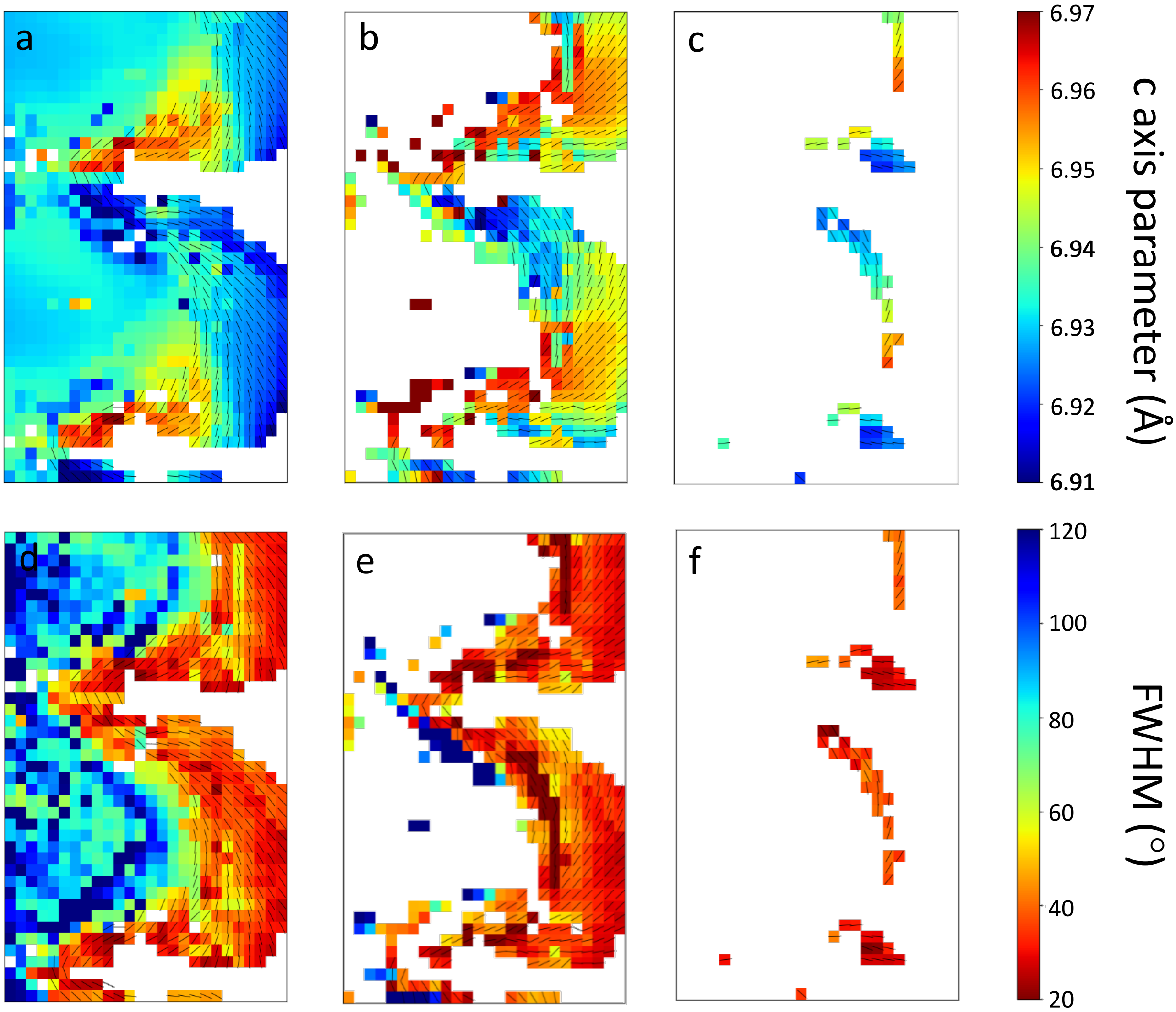

Supplementary Figure 19. **Synchrotron-based X-Ray Micro-diffraction (S-µXRD) maps of two serrations in longitudinal section (UALVP 53472).** The crystallographic c axis lattice and texture parameters of the three constituent crystallite populations within the tooth enamel are shown arranged by columns for population one (**a** and **d**), two (**b** and **e**) and three (**c** and **f**). Lines within each pixel indicate the average preferred apatite crystal orientation and hotter colours correspond to more highly textured regions (lower full-width half maxima). Orientation direction and FWHM in d, e, and f were used to calculate average values illustrated in main text Figure 4p.

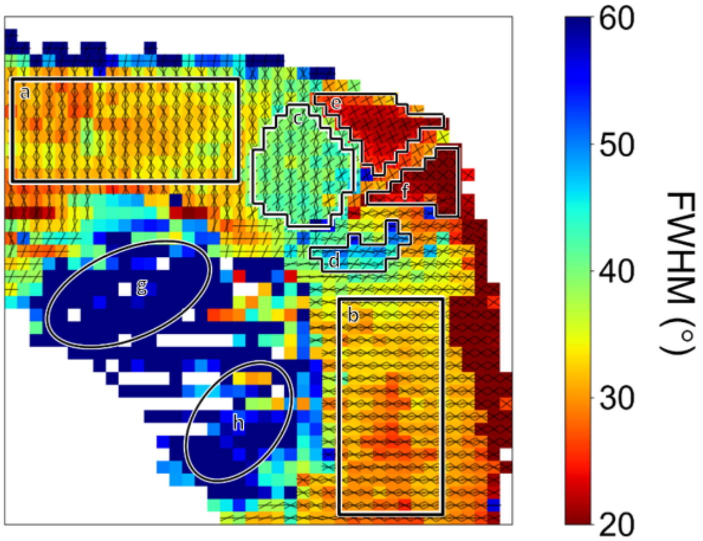

Map Regions of Interest	FWHM (°)
a and b	31.51 ± 2.36
c and d	42.02 ± 3.95
e and f	24.62 ± 6.70
g and h	81.35 ± 30.28

Supplementary Figure 20. **Synchrotron-based X-Ray Micro-diffraction (S-µXRD) map of a horizontal section through a serration and the surrounding enamel (UALVP 60554) with statistical comparisons of Full Width Half Maxima (FWHM) of enamel on- and off-serration.** Heat map (left) is derived from the same region as in main text Fig. 4q. Two-tailed t-tests were conducted to compare enamel and dentine regions on either side of the serration. Laterally symmetrical regions (e.g., a and b) were grouped together as single samples and compared with other regions. Mean Full Width Half Maxima (FWHM) values for grouped regions and associated standard deviations are summarised in the table (right). Statistical comparisons between each region were all statistically significant ($p<0.001$), indicating that each region contained apatite crystallite populations that significantly differed in terms of their Full Width Half Maxima (FWHM), which is a measure of the degree of variation around the principal orientations (small lines in each pixel) derived from S-µXRD analyses. See Extended Data 5 for full statistical analysis outputs and raw data for each grouping. Abbreviations: FWHM Full Width Half Maximum. Heat map colours indicate magnitude of FWHM, with hotter colours indicating lower FWHM values and therefore more highly ordered crystallites around a preferred (principal) orientation. Note that the lowest FWHM values are concentrated towards the serration tip (top right corner of map), corresponding to the wavy enamel identified under SEM.

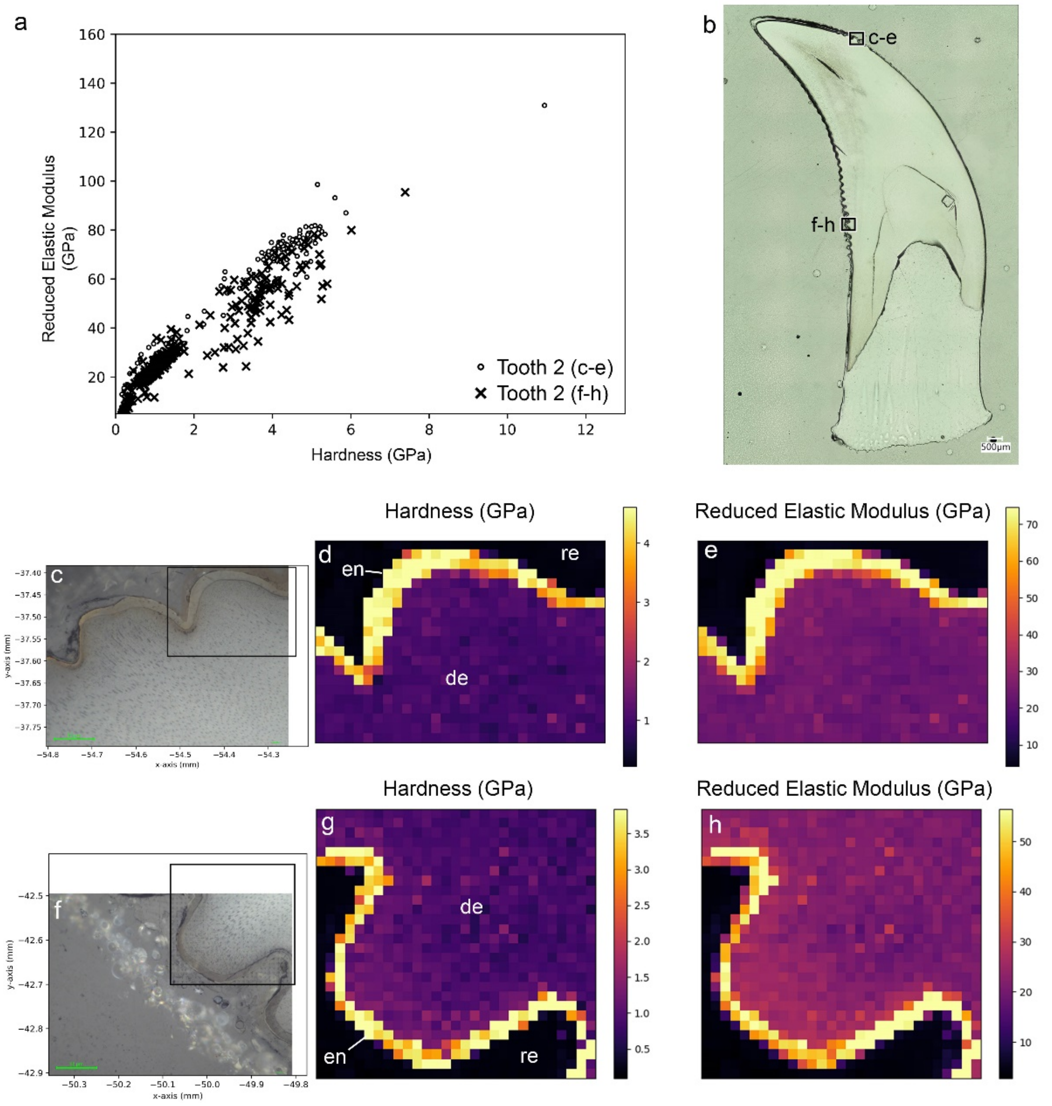

Supplementary Figure 21. **Nanoindentation analysis of *Varanus komodoensis* J94036-2. a** Enamel and dentine indentation hardness plotted against the indentation elastic modulus. **b** Coaxial light image of the polished longitudinal thick section of J94036-2 used for the nanoindentation tests. **c** Region of interest imaged in the nanoindenter prior to the experiment. **d** Heat map of Hardness (GPa) in the region indented in c. **e** Heat map of Reduced Elastic Modulus (GPa) in the region indented in c. **f** Region of interest imaged in the nanoindenter prior to the experiment. **g** Heat map of Hardness (GPa) in the region indented in f. **h** Heat map of Reduced Elastic Modulus (GPa) in the region indented in f. Abbreviations: de dentine, en enamel, re resin. See Methods for experimental parameters for nanoindentation tests.

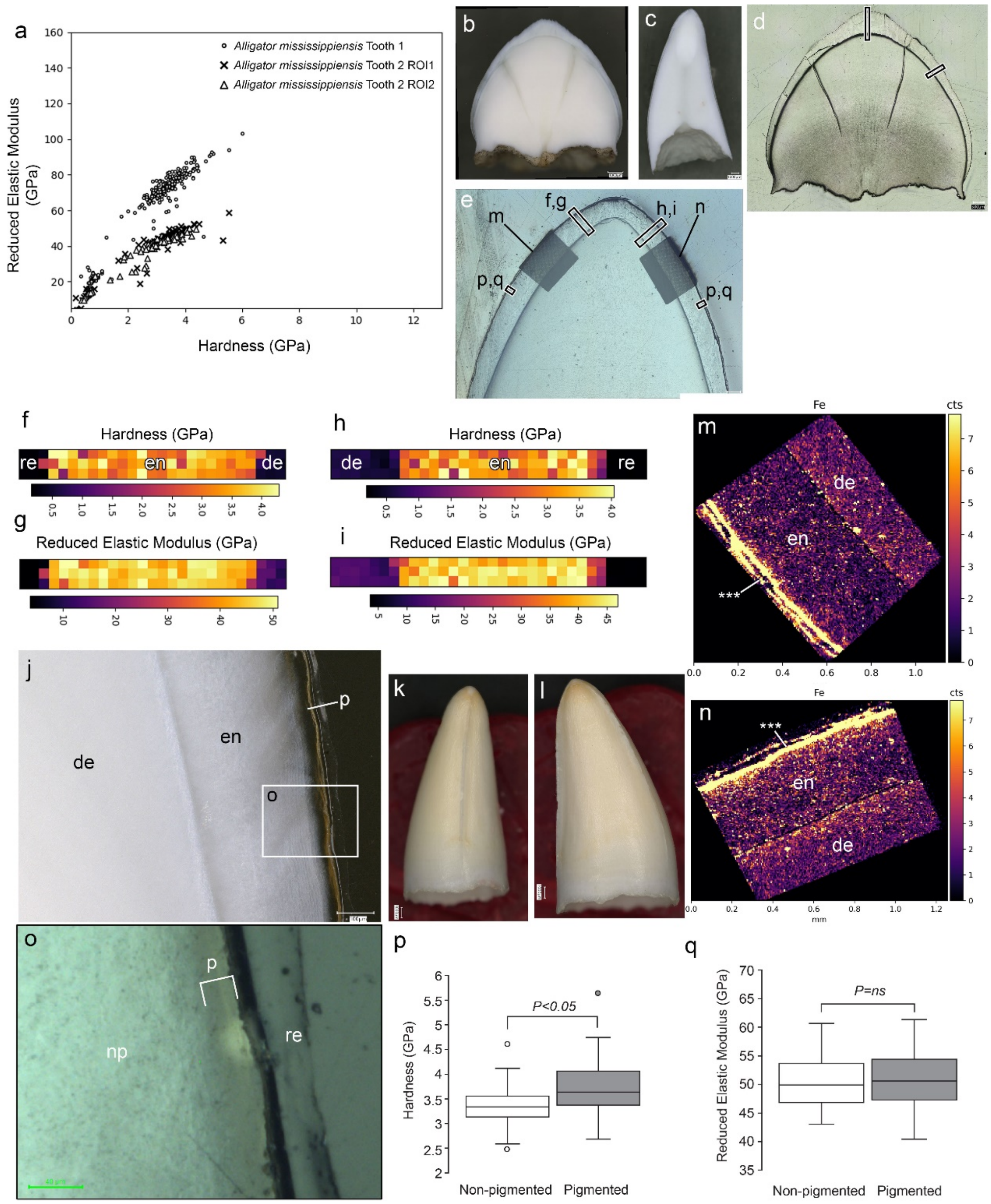

Supplementary Figure 22. **Comparisons of hardness and reduced elastic moduli in two *Alligator mississippiensis* teeth, with a focus on the iron-enriched enamel layers. a** Enamel and dentine hardness plotted against the reduced elastic modulus. **b** Polished thick section of *A. mississippiensis* "Tooth 1", a shed posterior tooth. **c** Polished thick section of *A. mississippiensis* "Tooth 2", a shed anterior tooth. **d** Coaxial light image of "Tooth 1" showing positions of nanoindentation regions of interest. **e** Tip of tooth crown from "Tooth 2" under coaxial light, showing ablation marks from LA-ICP-MS experiments, as well as positions of nanoindentation regions. **f** Heat map of hardness and **g** Elastic modulus, measured along the entire enamel and a portion of the dentine of "Tooth 2". **h** Heat map of hardness and **i** Reduced elastic modulus in a second region of interest from "Tooth 2". **j** High-magnification image of the enamel in polished thick section of "Tooth 2", showing the thickness of the enamel and position of an outer orange-coloured enamel layer found around the cutting-edge enamel. **k**

Distal view of "Tooth 2". **l** Lingual view of "Tooth 2" showing lack of any obvious pigmentation along the cutting edges under plain lighting. Orange colouration is most obvious in polished thick sections. **m, n** Raw LA-ICP-MS maps for iron along the enamel and dentine of "Tooth 2" showing the presence of an iron-enriched outer enamel layer in the same positions as the orange-coloured region in j. **o** Region of interest along the outer layers of enamel in j where more detailed nanomechanical testing was undertaken to directly compare the mechanical properties of pigmented and non-pigmented enamel. **p** Welch test of hardness of pigmented and non-pigmented enamel in the same tooth measured via nanoindentation [n= 38 (non-pigmented), 36 (pigmented) indents within 1 tooth]. Central lines represent medians (3.33, 3.63), upper and lower bounds of boxes represent lower (3.14, 3.38) and upper quartiles (3.55, 4.06), minima (2.47, 2.68) and maxima (4.6, 5.63) for non-pigmented and pigmented enamel respectively. p-value (two-tailed) was 0.0058. (Extended Data 4). **q** Pooled variance t-test comparing the indentation elastic moduli of pigmented and non-pigmented enamel in the same tooth measured via nanoindentation [n= 38 (non-pigmented), 36 (pigmented) indents within 1 tooth]. Central lines represent medians (49.88, 50.57), upper and lower bounds of boxes represent lower (46.70, 47.28) and upper quartiles (53.67, 54.41), minima (43.04, 47.28) and maxima (60.70, 61.34) for non-pigmented and pigmented enamel respectively. p-value (two-tailed) was 0.45 (Extended Data 4). Abbreviations: de dentine, en enamel, np, non-pigmented enamel, p pigmented enamel, re resin. Asterisks indicate position of iron-enriched, pigmented enamel.

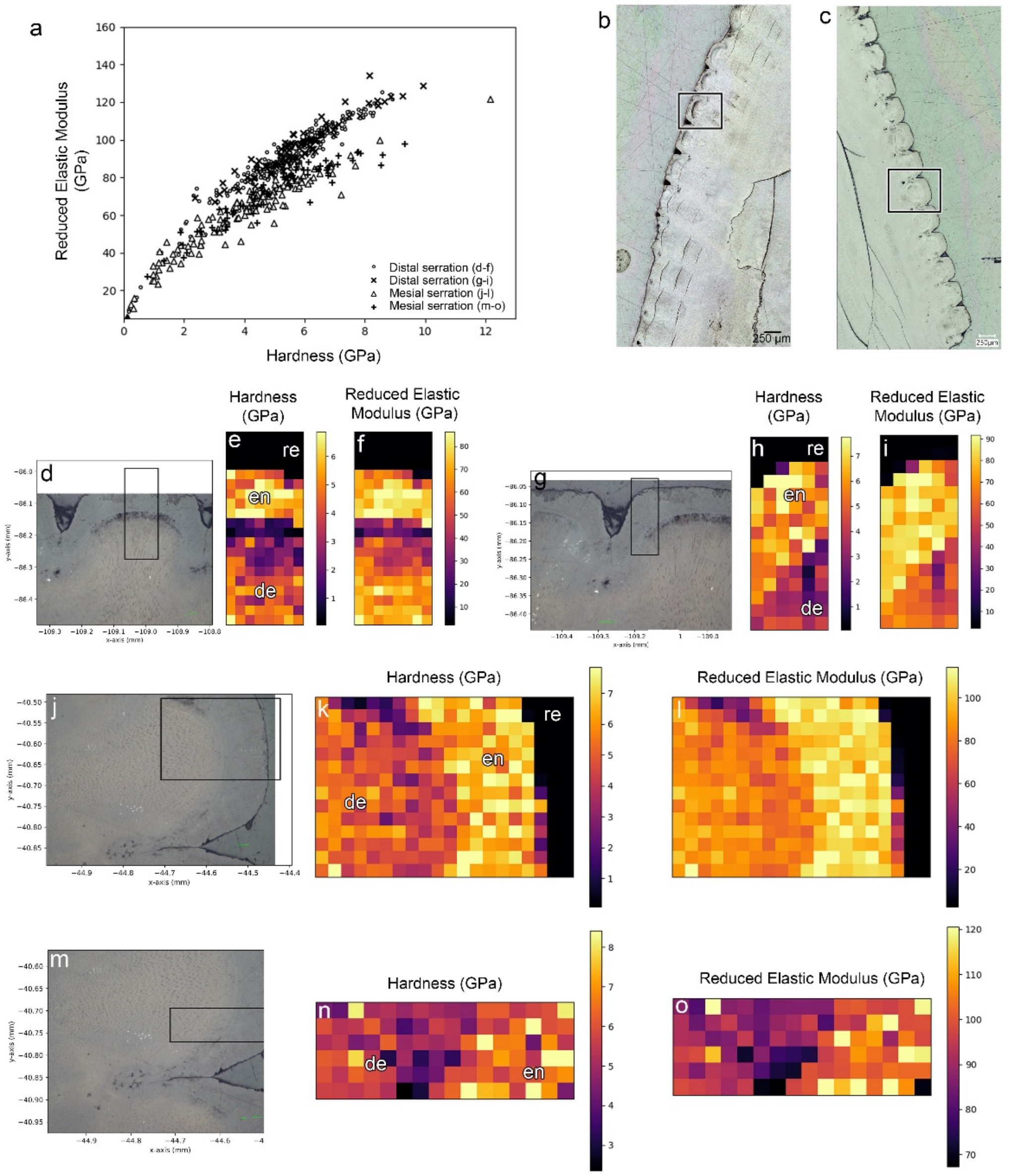

Supplementary Figure 23. **Nanoindentation analysis of a tyrannosaurid tooth (UALVP 60555). a** Enamel and dentine hardness plotted against the reduced elastic modulus. Note the lack of separation between dentine and enamel measurements and the higher magnitude of both metrics compared with data from extant reptile teeth. **b** Wholeview image of mesial serrations in polished thick section under coaxial light, showing region of interest in nanoindentation experiments. **c** Wholeview image of distal serrations under coaxial light, showing region of interest for nanoindentation experiments. **d** Region of interest along a mesial serration imaged in the nanoindenter prior to the experiment. **e** Heat map of hardness measured from first region of interest along the middle of the mesial serration. Note the similarity between the enamel and underlying dentine. **f** Heat map of reduced elastic modulus in same region. **g** Second region of interest along mesial serration imaged in the nanoindenter prior to the experiment. **h** Heat map of hardness measured from first region of interest along the middle of the mesial serration. Note the similarity between the enamel and underlying dentine. **i** Heat map of reduced elastic modulus in same region. **j** Third region of interest along distal serration imaged in the nanoindenter prior to the experiment. **k** Heat map of hardness of enamel and dentine measured

in third region of interest. **l** Heat map of reduced elastic modulus in same region. **m** Fourth region of interest along a distal serration imaged in the nanoindenter prior to the experiment. **n** Heat map of hardness of enamel and dentine measured in the fourth region of interest. **o** Heat map of the reduced elastic modulus in the same region. Abbreviations: de dentine, en enamel, re resin.

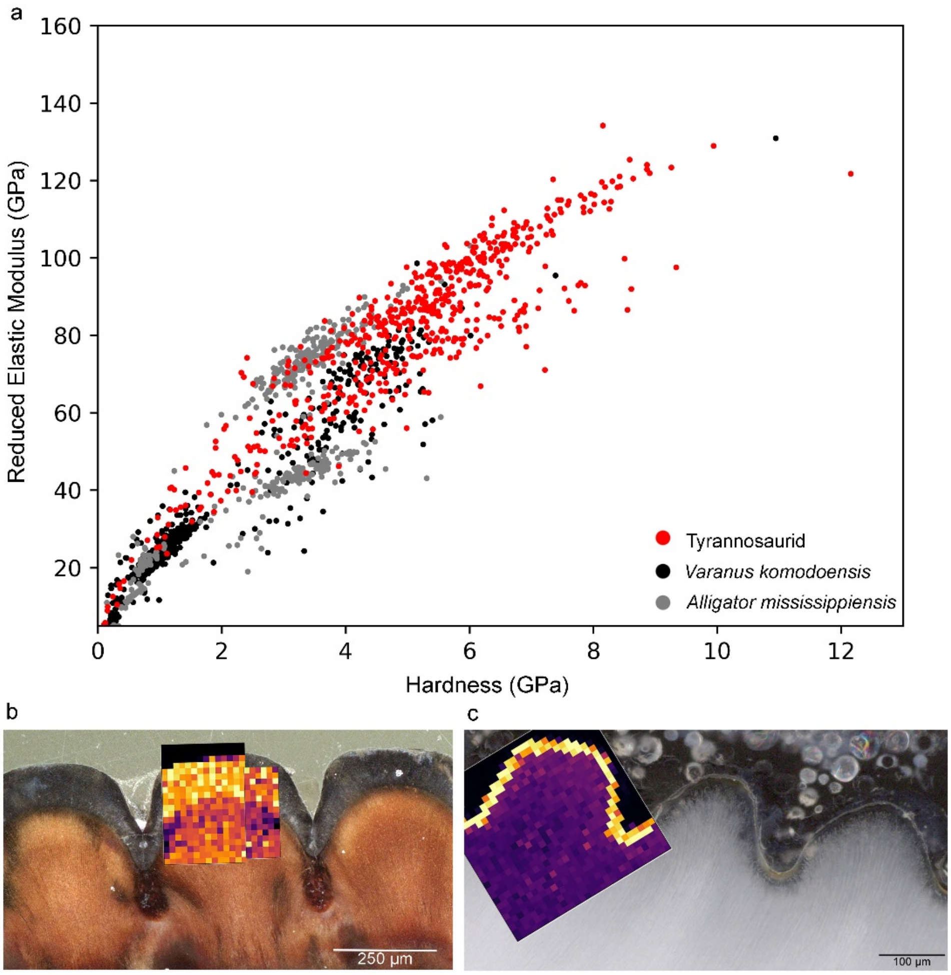

Supplementary Figure 24. **Comparisons of indentation hardness and reduced elastic moduli of extant *Varanus komodoensis*. *Alligator mississippiensis*, and tyrannosaurid teeth. a** Combined plot of nanomechanical properties of tyrannosaurid (red), *Varanus komodoensis* (black), and *Alligator mississippiensis* enamel and dentine. Tyrannosaurid tooth indents yielded higher hardness and elastic moduli compared with equivalent regions in the two extant reptiles. **b** Two hardness heat maps (Supplementary Fig. 18k, n) superimposed on polished thick section of the tyrannosaurid tooth Note the more subtle differences in hardness between the dentine and enamel in the fossil tooth, due to chemical and structural alterations to the two tissues. **c** Comparisons with a relative hardness map of *Varanus komodoensis* (Supplementary Fig. 17g). Note the stark contrast between the enamel and dentine. These comparisons demonstrate the impact of fossilization on the mechanical properties of tyrannosaurid enamel and dentine.

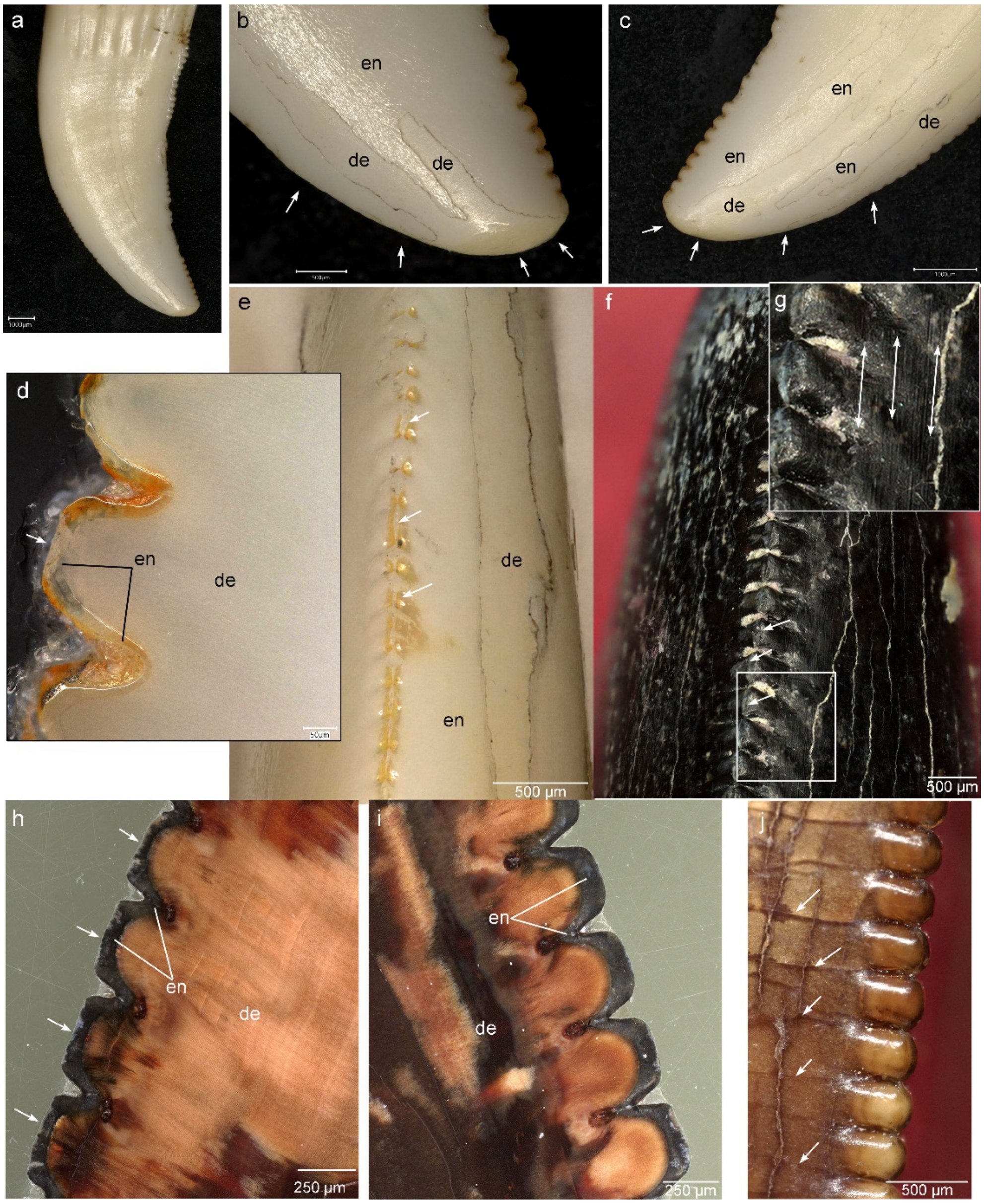

Supplementary Figure 25. **Comparisons of tooth wear in *Varanus komodoensis* and tyrannosaurid teeth.** **a** Labial view of a worn *V. komodoensis* tooth (MoLS X263-2). **b** Closeup of worn tooth tip in labial view, showing large-scale spalling of enamel and exposure of the dentine, except along the serrations. **c** Closeup of lingual surface of tooth tip, showing similar spalling and flaking of enamel, exposing large portions of the crown dentine. **d** Polished thick section through distal denticles of another *V. komodoensis* tooth (J94036-2), showing small-scale wear along the serration tips and subtle loss of pigmentation, beginning at highest point of serration. **e** Worn mesial denticles of MoLS X263-2 showing subtle apical wear of each denticle, compared with the large-scale flaking of enamel in other regions. **f** Closeup of partially worn serrations in a tyrannosaurid tooth (UALVP 60555), showing the gradual wear of the cutting surface. **g** Closeup of apico-basal wear striations, indicating direction of wear (double arrowheads). **h** Same worn mesial serrations in UALVP 60555 in polished thick section, showing gradual wear of serration enamel. **i** Comparison with relatively unworn distal serrations of the same tyrannosaurid tooth. **j** Closeup image of enamel surface of another

tyrannosaurid tooth (UALVP 60553) under reflected light, highlighting many (post-mortem) cracks (arrows) through the columnar enamel of the crown, and the lack of these cracks within the wavy enamel of the serrations. Abbreviations: de dentine, en enamel. Unless otherwise indicated, arrows indicate worn surfaces of teeth.

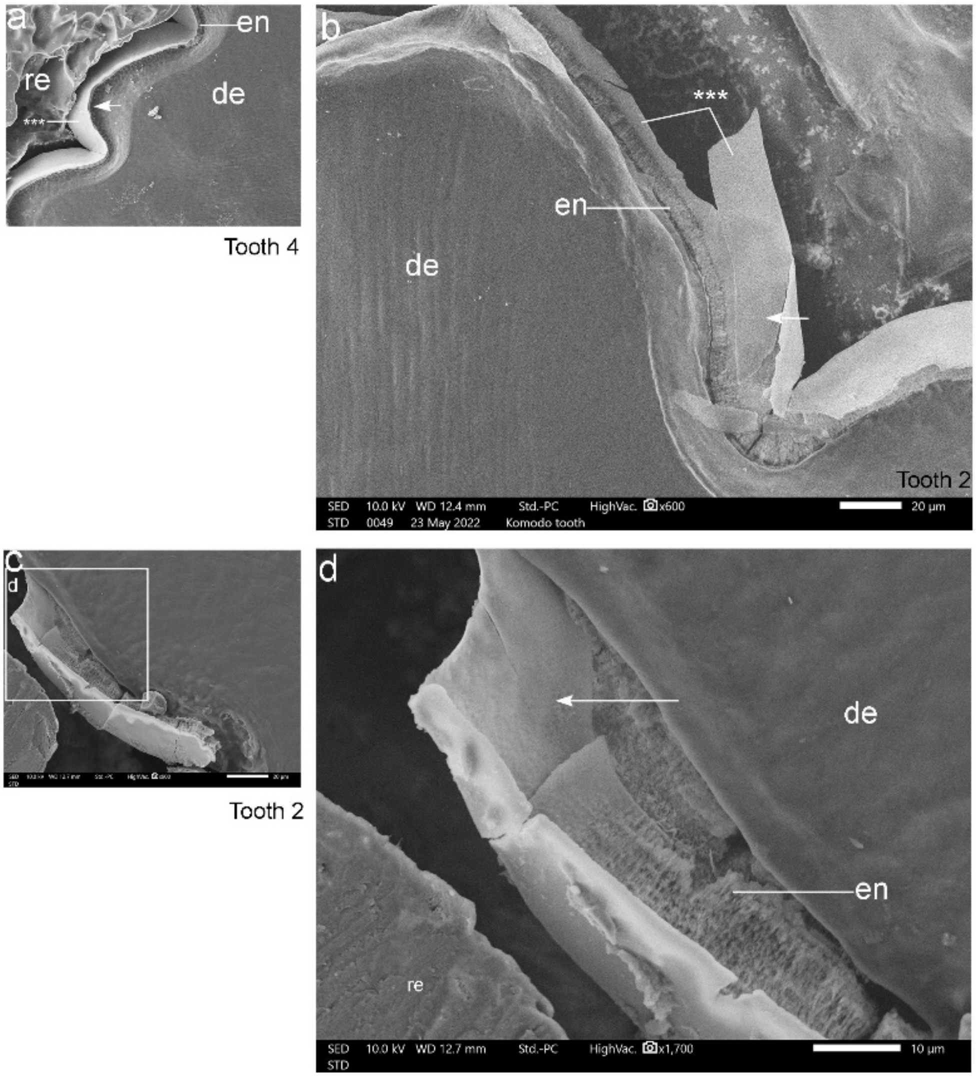

Supplementary Figure 26. **Scanning Electron Microscope (SEM) imaging of acid-etched serrations in *Varanus komodoensis*, showing acid-resistance of the outer iron-rich coating.** **a** Mesial serrations of a *V. komodoensis* tooth under SEM following a 30-second immersion in 1M HCl. The resistance of the outer iron-rich coating (asterisks) lead to the formation of an overhang (arrow), created by the dissolution of the underlying enamel (J94036-4). **b** Similar feature after 30 seconds of etching in 1M HCl. The underlying enamel has nearly completely dissolved away, leaving an unsupported outer shell of the iron-rich material (asterisks), which collapsed under its own weight (arrow). **c** Serration in J94036-2 showing nearly complete dissolution of enamel after 30 seconds of 1M HCl etching. **d** Higher magnification image showing dissolution of enamel and preservation of the outer iron-rich coating as an unsupported shell over the dentine (arrow). Abbreviations: de dentine, en enamel, re resin.

Supplementary Table 1. **Survey of tooth pigmentation in reptiles.**

Taxon	Species	Specimen no.	Orange pigment?	Location on tooth	Number of teeth	Source
Squamata: Varanidae *Varanus komodoensis*		NHMUK 1934.9.2.1 (skull + mandibles)	Y	On tooth tips and serrations	All teeth	This study
		NHMUK 1985. 1226 (Skull)	Y	On tooth tips and serrations	All teeth	This study
		MoLS X-263 (three isolated teeth)	Y	On tooth tips and serrations	All teeth	This study
		J94036 (five isolated teeth)	Y	On tooth tips and serrations	All teeth	This study
		AMNH 37909 (skull + mandibles)	Y	On tooth tips and serrations	All teeth	This study
		AMNH 37913 (skull + mandibles)	Y	On tooth tips and serrations	All teeth	This study
		AMNH 37911 (skull + mandibles)	Y	On tooth tips and serrations	Faint on erupted teeth, obvious on unerupted	This study
		AMNH 37910 (skull)	Y	On tooth tips and serrations	spotty and faint in areas	This study
		AMNH 37879 (partial skull/mandible; chimera?)	Y	On tooth tips and serrations	All teeth	This study
		AMNH 74606 (skull + mandibles)	Y	On tooth tips and serrations	All teeth	This study
		AMNH 37912 (skull + mandibles)	Y	On tooth tips and serrations	fainter on upper tooth row, especially faint on smaller teeth	This study
		AMNH 37908 (skull + mandibles)	faint	On serrations	All teeth	This study
		AMNH 109498 (skull + mandibles)	faint	Only on unerupted serrations. Some erupted apices are very orange.	May be due to preparation, skeleton is very green.	
		London Zoo (ZSL) (lower jaw, soft tissue preserved in ethanol)	Y	On tooth tips and serrations	All teeth	This study
Squamata: Varanidae	*Varanus salvator*	NHMUK 1972.2160	Y	On tooth carinae	Middle of tooth row	This study
		NHMUK 64 .9-2 77	N	N/A	N/A	This study
		NHMUK 1872 2161	N	N/A	N/A	This study
		AMNH 141148	Y	tips only	All teeth	This study
		AMNH 141155	Y	tips only	All teeth	This study
		AMNH 49230	faint	tips only	All teeth	This study
		AMNH 28356	N	N/A	N/A	This study
		AMNH 57765	N	N/A	N/A	This study
		AMNH 142471	N	N/A	N/A	This study
		AMNH 147174	N	N/A	N/A	This study
Squamata: Varanidae	*Varanus indicus*	NHMUK 1932. 4. 192	Y	On tooth tips, distal carinae, and serrations	Serrated, mid-tooth row teeth, carinated anterior teeth	This study

		AMNH 142623	Y	tips only	All teeth	This study
		AMNH 114512	Y	tips and upper carinae	All teeth	This study
		AMNH 58389	N	N/A	Enamel is transparent, may be due to preparation	This study
		AMNH 114513 (mandible only)	N	N/A	All teeth	This study
Squamata: Varanidae	*Varanus gouldii*	BM (NHMUK) 1983	N	N/A	N/A	N/A
		AMNH 74810 (skull + mandibles)	N	N/A	N/A	This study
		AMNH 82819 (skull + mandibles)	N	N/A	Enamel is transparent, may be due to preparation	This study
		SAMA R27031 (skull + mandibles)	faint	Very faint, on replacement teeth	Only on replacement teeth	This study
		SAMA R27032 (skull + mandibles)	N	N/A	N/A	N/A
		SAMA R 27030 (skull + mandibles)	N	N/A	N/A	N/A
		SAMA R52606 (disarticulated skull + mandibles)	faint	Very faint, on functional teeth	Functional teeth only, and very faint	This study
		SAMA R32627	N	N/A	N/A	N/A
Squamata: Varanidae	*Varanus salvadorii*	AMNH 59873 (skull + mandibles)	Y	On tooth tips and serrations	All teeth	This study
		FLMNH 70028 (skull only)	Y	On tooth tips and serrations	All teeth	This study
		FLMNH 70029	Y	On tooth tips and serrations	All teeth	This study
Squamata: Varanidae	*Varanus varius*	AMNH 73361 (skull + mandibles)	Y	On tooth tips and serrations	All teeth	This study
		AMNH 28698 (skull + mandibles)	N	N/A	May be due to preparation, enamel is transparent	This study
		FLMNH 55476	Y	On tooth tips and serrations	All teeth	This study
		SAMA R40022	N	N/A	N/A	N/A
		SAMA R27033	N	N/A	N/A	N/A
Squamata: Varanidae	*Varanus giganteus*	SAMA R33352	Y	On tooth tips and serrations of replacement teeth and functional teeth	All teeth, sometimes worn away on functional teeth	This study
		SAMA R27034	Y	On tooth tips and serrations of functional teeth	All teeth	This study
Squamata: Varanidae	*Varanus rosenbergi*	SAMA R13829	Y	On tooth tips and serrations	All teeth	This study
		SAMA R10295	N	N/A	N/A	N/A
Squamata: Varanidae	*Varanus tristis orientalis*	SAMA R3224.C	N	N/A	N/A	N/A

Squamata: Varanidae	*Varanus griseus*	NHMUK 1974. 2481	N	N/A	N/A	N/A
		NHMUK 1974. 2482	N	N/A	N/A	N/A
		NHMUK 1974. 2483	N	N/A	N/A	N/A
		NHMUK 19201.20.722	N	N/A	N/A	N/A
		NHMUK 19201.20.722	N	N/A	N/A	N/A
Squamata: Varanidae	*Varanus gilleni*	SAMA R14856.B	N	N/A	N/A	N/A
		SAMA R67632	faint	Very faint, on unserrated cutting edges	All teeth	This study
		SAMA R1758	N	N/A	N/A	N/A
Squamata: Varanidae	*Varanus bengalensis*	NHMUK 1931.1.10.2	N	N/A	N/A	N/A
		MoLS X38 (small)	N	N/A	N/A	N/A
		SAMA R15716.B	N	N/A	N/A	N/A
Squamata: Varanidae	*Varanus albigularis*	NHMUK 1903.4.24.35	N	N/A	N/A	N/A
Squamata: Varanidae	*Varanus niloticus*	NHMUK 97.5.31.2 (large)	N	N/A	N/A	N/A
		NHMUK 2018.2784 (small)	N	N/A	N/A	N/A
		MoLS X174 (small)	N	N/A	N/A	N/A
		MoLS X266 (large)	N	N/A	N/A	N/A
Squamata: Varanidae	*Varanus exanthematicus*	NHMUK 1920.1.20.3755 (small)	N	N/A	N/A	N/A
		NHMUK 1920.1.20.3660 (larger)	N	N/A	N/A	N/A
		SAMA R66662	N	N/A	N/A	N/A
Squamata: Varanidae	*Varanus caudolineatus*	SAMA R35574	N	N/A	N/A	N/A
Squamata: Varanidae	*Varanus eremius*	SAMA R49942	N	N/A	N/A	N/A
		SAMA R45497	N	N/A	N/A	N/A
Squamata: Varanidae	*Varanus storri*	SAMA unnumbered (“SVL121mm”)	N	N/A	N/A	N/A
		SAMA unnumbered (“SVL115mm”)	N	N/A	N/A	N/A
Squamata: Varanidae	*Varanus scalaris “pellewensis”*	SAMA unnumbered (“SVL195mm”)	N	N/A	N/A	N/A
Squamata: Helodermatidae	*Heloderma sp.*	MoLS X185	N	N/A	N/A	N/A
Squamata: Helodermatidae	*Heloderma horridum*	NHMUK 1960.830 (larger)	N	N/A	N/A	N/A
		(NHMUK) II.1A (smaller)	N	N/A	N/A	N/A
Squamata: Helodermatidae	*Heloderma suspectum*	NHMUK 1911.6.9.1	N	N/A	N/A	N/A
Squamata: Agamidae	*Japalura polygonata*	N/A	Y	Along edges of triconodont teeth	Posterior triconodont teeth only	Ishiyama and Teraki (1992)

Squamata: Agamidae	Agamidae indet.	MoLS X68	N	N/A	N/A	N/A
Squamata: Teiidae	*Tupinambis* sp.	MoLS X259	N	N/A	N/A	N/A
Squamata: Iguanidae	Iguanidae indet.	MoLS X48	N	N/A	N/A	N/A

Supplementary Table 2. **Synchrotron experiment parameters**

	Beamline	Sample ID	Sample plane and thickness	Imaging technique	Beam spot size (X, Y, microns)	Incident energy (keV)	Detector type	Acquisition time per pixel (s)	Sample environment	Step size (microns)	Figure number
MM22284	DLS-B16	UALVP 53472 (tyrannosaurid tooth)	Sectioned along serrations and thinned to 200 microns	X-ray fluorescence	24 x 19	15.5	VORTEX 90EX	5	Ambient pressure and temperature	20	Not figured
MM22284	DLS-B16	UALVP 53472 (tyrannosaurid tooth)	Sectioned along serrations and thinned to 200 microns	X-ray diffraction	24 x 19	15.5	Imagestar diffraction detector, positioned 140mm from sample	20-30s	Ambient pressure and temperature	20	Fig. 6j
MM26050	DLS-B16	UALVP 53472 (tyrannosaurid tooth)	Sectioned along serrations and thinned to 200 microns	X-ray fluorescence	2.9 x 1.8	15.5	Imagestar diffraction detector, positioned 140mm from sample	5	Ambient pressure and temperature	10	Suppl. Fig. 12
MM26050	DLS-B16	UALVP 53472 (tyrannosaurid tooth)	Sectioned along serrations and thinned to 200 microns	X-ray diffraction	2.9 x 1.8	15.5	Imagestar diffraction detector, positioned 140mm from sample	60	Ambient pressure and temperature	10	Not figured
28-01-1286	ESRF-BM28	XM1 (extant *Osteolaemus tetraspis* tooth)	Sectioned along carina then thinned to ~200 microns	X-ray fluorescence	20 x 20	19	Ketek Silicon Drift Detector	5	Ambient pressure and temperature	20	Suppl. Fig. 6g-ji; Suppl. Fig. 7a, b
28-01-1286	ESRF-BM28	XM4 (extant *Crocodylus porosus* tooth)	Sectioned along carina then thinned to ~200 microns	X-ray fluorescence	20 x 20	19	Ketek Silicon Drift Detector	5	Ambient pressure and temperature	20	Fig. 4k-m; Suppl. Fig. 6k-n; Suppl. Fig. 7e-g
28-01-1286	ESRF-BM28	XM2 (UALVP 60546) Fossil crocodylian tooth-bulbous	Sectioned along carina then thinned to ~200 microns	X-ray fluorescence	20 x 20	19	Ketek Silicon Drift Detector	5	Ambient pressure and temperature	20	Suppl. Fig. 7c,d
28-01-1286	ESRF-BM28	XM5 (UALVP 60550) Fossil crocodylian tooth-conical	Sectioned along carina then thinned to ~200 microns	X-ray fluorescence	20 x 20	19	Ketek Silicon Drift Detector	5	Ambient pressure and temperature	20	Fig. 4o-r; Suppl. Fig. 7h-j
LS-3074	ESRF-ID21	MoLS X-263 (developing *Varanus komodoensis* tooth)	Tooth tip and serrations sectioned longitudinally. Thickness >1000 microns	X-ray fluorescence	0.8 x 0.2	9.8	Sirius SD silicon drift detector	0.2	Vacuum	5	Fig. 2b

LS-3074	ESRF-ID21	MoLS X-263 (developing *Varanus komodoensis* tooth)	Tooth tip and serrations sectioned longitudinally. Thickness >1000 microns	X-ray fluorescence	0.8 x 0.2	9.8	Sirius SD silicon drift detector	0.2	Vacuum	0.5	Fig. 2c-f; Suppl. Fig. 2b, c
LS-3074	ESRF-ID21	MoLS X-263 (developing *Varanus komodoensis* tooth)	Horizontal section through serrations close to tooth tip ("XS1"). Thickness ~300 microns	X-ray fluorescence	0.8 x 0.2	9.8	Sirius SD silicon drift detector	0.2	Vacuum	0.5	Fig. 2g, i
LS-3074	ESRF-ID21	MoLS X-263 (developing *Varanus komodoensis* tooth)	Horizontal section through serrations slightly farther from tooth tip ("XS2"). Thickness ~300 microns	X-ray fluorescence	0.8 x 0.2	9.8	Sirius SD silicon drift detector	0.2	Vacuum	0.5	Suppl. Fig. 2a
LS-3074	ESRF-ID21	MoLS X-263 (developing *Varanus komodoensis* tooth)	Tooth tip and serrations sectioned longitudinally. Thickness >1000 microns	X-ray fluorescence	0.8 x 0.2	9.8	Sirius SD silicon drift detector	0.2	Vacuum	0.5	Suppl. Fig. 2d
LS-3074	ESRF-ID21	MoLS X-263 (developing *Varanus komodoensis* tooth)	Tooth tip and serrations sectioned longitudinally. Thickness >1000 microns	X-ray fluorescence	0.8 x 0.2	9.8	Sirius SD silicon drift detector	0.2	Vacuum	0.5	Suppl. Fig. 2f
LS-3074	ESRF-ID21	J94036-1 (functional *Varanus komodoensis* tooth)	Sectioned along distal serrations. Thickness ~300 microns	X-ray fluorescence	0.8 x 0.2	9.8	Sirius SD silicon drift detector	0.2	Vacuum	0.5	Suppl. Fig. 2e
LS-3074	ESRF-ID21	UALVP 60553 (Tyrannosaurid premaxillary tooth)	Horizontal section through serrations. Thickness ~300 microns	X-ray fluorescence	0.8 x 0.2	9.8	Sirius SD silicon drift detector	0.2	Vacuum	5	Suppl. Fig. 12c-f
LS-3093	ESRF-BM28	UALVP 60554 (Tyrannosaurid tooth)	Horizontal section through distal serration. Thickness <200 microns	X-ray diffraction	10 x 10	15	MAR detector		Ambient pressure and temperature	10	Fig. 6k
SP-35162	DLS-I18	XM4 (extant *Crocodylus prorosus* tooth)	Sectioned along carina then thinned to ~200 microns	Fe-XANES	2 x 2	6.96-7.25 at 0.5 eV increments	VORTEX 90EX		Ambient pressure and temperature	N/A: point measurements	Suppl. Fig. 9

SP-35162	DLS-I18	MoLS X-263 (developing *Varanus komodoensis* tooth)	Tooth tip and serrations sectioned longitudinally. Thickness >1000 microns	Fe-XANES	2 x 2	6.96-7.25 at 0.5 eV increments	VORTEX 90EX	Ambient pressure and temperature	N/A: point measurements	Suppl. Fig. 9
SP-35162	DLS-I18	UALVP 56917 (Beaver incisor *Castor canadensis*)	Transverse section through incisor. Thickness >1000 microns	Fe-XANES	2 x 2	6.96-7.25 at 0.5 eV increments	VORTEX 90EX	Ambient pressure and temperature	N/A: point measurements	Suppl. Fig. 9

Supplementary Table 3. **Raw data and t-tests comparing Full Width Half Maxima (FWHM) between groups of pixels along Synchrotron X-Ray Microdiffraction map of a cross-section of a tyrannosaurid tooth (UALVP 60554).** See Supplementary Figure 20 for locations of pixel groups compared in the t-tests.

Texture Map Regions of Interest FWHM (°)			
a&b	c&d	e&f	g&h
30.63552	37.5755	26.98812	64.83453485
30.83838	39.46286	25.80561	65.69360561
31.40029	39.11273	26.08861	90.4454871
29.84717	40.53996	26.54744	84.70670306
27.1777	40.91653	26.94351	89.10048608
28.98906	38.47787	30.63194	76.96497919
28.51811	39.23879	32.2118	85.82380798
34.66561	41.07946	24.28709	69.07772127
32.00967	39.81879	23.34593	80.373031
31.50769	38.69278	22.79379	84.16122392
30.41472	39.05854	22.34533	129.6730904
32.71155	40.71531	22.17589	69.40055199
30.77654	39.77905	22.6724	69.47220178
33.35737	40.44739	25.19717	86.62575163
36.1079	39.29386	30.60587	66.96188075
37.62327	39.87588	24.14811	79.02010193
36.29836	39.69286	21.78451	66.42585058
30.63668	36.03911	21.43602	60.61103645
30.97945	38.99597	21.65453	66.8162944
31.99246	42.76202	21.49467	57.05389984
29.72907	41.37329	20.78477	68.95792363
31.0184	39.18784	21.81223	65.74210875
27.67381	40.98886	20.73461	67.38496565
28.83851	39.04711	19.63156	64.74683635
32.2383	40.03613	25.02086	70.92925946
29.67616	39.4269	22.85535	72.67688184
32.75928	40.60191	21.59216	62.5488435
32.81389	40.25894	21.53767	55.78373149
32.85912	41.57975	21.84444	63.30980737
33.45334	39.19877	23.12659	63.69053062
33.73095	41.4113	26.44471	57.89877949
32.70756	42.21388	24.09681	87.40209339
33.67137	41.36601	22.91708	63.84013483
34.53834	42.9188	22.83574	79.98946173
26.7042	41.0857	24.80651	83.61434374
31.04719	54.50489	25.28636	108.9772669
30.7795	40.89648	24.60791	96.06851485
29.64927	36.83297	24.22798	109.3007371
31.02902	49.93023	20.08174	129.9263175

26.96261	48.93998	17.97958	136.4081084
27.88082	49.17738	19.36526	150.1660161
32.41844	50.83739	17.65716	158.680189
33.02665	46.61693	19.45749	126.0110593
32.6967	47.69364	40.03613	129.4691315
31.466	49.35957	38.68729	187.6947108
31.95758	43.96071	45.2048	75.27919445
30.88012	44.48063	43.67554	66.11483998
33.02115	47.25976	56.49937	71.43773102
32.28703	41.84222	26.7245	66.22884047
31.89926	43.78043	28.9223	60.57936233
33.12307	44.56168	21.38406	67.37434567
31.43743		19.17402	77.62651535
29.85115		19.69854	62.1123447
27.52574		19.88835	73.63712139
31.86061		19.53575	81.97308696
30.09659		20.60886	91.84890998
29.64443		21.71921	89.0202841
37.30712		21.5077	196.7333175
29.53732		20.86496	101.8719282
29.60579		19.72284	73.14055241
29.49519		18.81789	166.2704942
30.65006		22.24328	145.9124038
30.46916		24.31621	155.1800065
32.13583		24.37108	119.0099132
31.7441		23.13341	117.9526385
32.3174		28.19019	74.82119242
32.33816		21.20642	135.4808825
33.64301		19.9725	89.04230935
32.95549			65.10466854
32.46162			77.45173245
30.61391			75.48908936
30.64329			94.6282495
28.61975			103.7875888
30.90492			83.60246349
39.27408			73.73484315
35.04617			70.85960285
32.6555			56.0572762
30.99087			74.49234652
30.72331			69.66338461
31.11223			68.64146259
30.88685			56.97594278
31.9924			153.3188004
32.67535			105.7445842
33.32845			71.80374991
34.71182			57.99778199

35.50597	73.36067078
33.84344	61.73589482
33.26174	55.74089664
34.74539	52.07686241
32.63335	55.06741596
32.35066	58.48704187
37.4998	56.07757977
35.58005	50.00634873
31.91991	58.79596611
31.38643	56.33316626
31.56786	72.26567503
31.81439	68.38848363
31.11706	61.43484998
32.41338	56.00916891
33.9299	87.07888562
36.78346	124.6973433
38.02	142.5387123
32.12691	98.14445771
32.66507	56.95271113
32.62382	77.70343935
34.71871	77.12323726
32.33996	72.16583886
32.96776	74.53774394
33.48071	130.4007644
31.96605	86.47088757
33.05472	76.10859085
31.8812	82.875281
32.71764	77.53248657
33.8654	55.25430474
34.10232	72.9317064
33.73815	54.49742168
35.05692	48.55430359
34.78839	50
34.78791	48.92449131
27.89363	136.9666746
31.2695	60.8237637
30.41914	88.24870714
32.12849	60.16957243
30.26087	77.36256188
32.30746	58.7159246
31.44831	41.65674023
31.17712	46.55204817
31.92462	48.85095483
29.92091	47.91452553
33.90986	81.62069319
33.73609	72.3283254

33.38931		73.39130132	
35.8216		71.75964201	
32.36117		56.57114429	
33.19498		45.02813276	
32.26126		43.37960456	
33.67511			
32.4121			
34.76677	t-Test: Paired Two Sample for Means		
34.55366			
35.09332		*a&b*	*c&d*
33.57598	Mean	31.54949457	42.01854
33.36832	Variance	5.357912839	15.56853
33.2925	Observations	51	51
33.49851	Pearson Correlation	-0.03026016	
31.40847	Hypothesized Mean Difference	0	
30.75433	df	50	
34.27359	t Stat	-16.1318104	
34.92846	P(T<=t) one-tail	9.28528E-22	
33.59485	t Critical one-tail	2.403271917	
32.35219	P(T<=t) two-tail	1.85706E-21	
32.79925	t Critical two-tail	2.677793271	
32.08799			
30.83356			
29.84179			
33.20698	t-Test: Paired Two Sample for Means		
32.47143			
32.29935		*a&b*	*e&f*
32.55205	Mean	31.54949457	25.66661
33.65181	Variance	5.357912839	53.87276
29.206	Observations	51	51
31.74844	Pearson Correlation	0.089277111	
32.68525	Hypothesized Mean Difference	0	
30.67729	df	50	
32.30797	t Stat	5.604265274	
31.65157	P(T<=t) one-tail	4.47952E-07	
32.4841	t Critical one-tail	2.403271917	
32.45136	P(T<=t) two-tail	8.95905E-07	
34.31503	t Critical two-tail	2.677793271	
32.52253			
33.42891			
32.6751	t-Test: Paired Two Sample for Means		
32.19308			
31.07952		*a&b*	*g&h*
31.99403	Mean	31.54949457	84.53871
33.06766	Variance	5.357912839	859.4331

31.53607	Observations	51	51
32.79718	Pearson Correlation	-0.23166956	
32.5761	Hypothesized Mean Difference	0	
32.86211	df	50	
32.91815	t Stat	-12.640442	
32.31814	P(T<=t) one-tail	1.72149E-17	
32.22141	t Critical one-tail	2.403271917	
32.16172	P(T<=t) two-tail	3.44299E-17	
41.35128	t Critical two-tail	2.677793271	
32.53534			
32.94131			
32.49446	t-Test: Paired Two Sample for Means		
28.13928			
30.70342		*c&d*	*e&f*
32.01439	Mean	42.01853583	25.66661
32.00115	Variance	15.56852871	53.87276
32.18109	Observations	51	51
31.85859	Pearson Correlation	0.186226273	
30.406	Hypothesized Mean Difference	0	
29.45745	df	50	
26.93022	t Stat	15.24762629	
31.43345	P(T<=t) one-tail	9.74863E-21	
30.68749	t Critical one-tail	2.403271917	
32.08927	P(T<=t) two-tail	1.94973E-20	
29.47923	t Critical two-tail	2.677793271	
32.61311			
32.3305			
29.59163			
27.94777	t-Test: Paired Two Sample for Means		
27.53539			
33.68187		*c&d*	*g&h*
31.17925	Mean	42.01853583	84.53871
30.59376	Variance	15.56852871	859.4331
32.00578	Observations	51	51
30.24531	Pearson Correlation	0.643937189	
29.73829	Hypothesized Mean Difference	0	
28.73609	df	50	
31.59944	t Stat	-11.2694532	
31.72352	P(T<=t) one-tail	1.23877E-15	
31.52182	t Critical one-tail	2.403271917	
32.91879	P(T<=t) two-tail	2.47754E-15	
32.2449	t Critical two-tail	2.677793271	
31.24121			
28.33208			
26.54245	t-Test: Paired Two Sample for Means		

30.64139
30.91682
30.1179
30.38674
31.29053
27.59959
27.25952
25.84484
26.64409
31.53794
30.98966
28.95024
31.795
30.43983
26.72626
27.79138
26.92937
28.65705
27.14558
32.09973
29.03822
29.488
29.86786
28.94912
28.65879
29.16895
31.66953
32.73897
29.81219
30.17887
29.89908
29.35628
29.50499
31.75562
31.75901
30.23557
29.82187
29.2722
31.9138
29.3689
29.32036
31.00726
31.86719
29.46096
30.20396
30.00424

	e&f	*g&h*
Mean	24.61717828	90.63394
Variance	44.94877712	1114.396
Observations	68	68
Pearson Correlation	-0.07009537	
Hypothesized Mean Difference	0	
df	67	
t Stat	-15.7762449	
P(T<=t) one-tail	1.4976E-24	
t Critical one-tail	2.383302488	
P(T<=t) two-tail	2.9952E-24	
t Critical two-tail	2.651219685	

	**mean FWHM (°)**	**standard deviation**
**a&b**	31.506	2.363
**c&d**	42.019	3.946
**e&f**	24.617	6.704
**g&h**	81.353	30.279

27.85558
28.37494
25.22934
30.33343
30.56619
29.2866
27.24718
28.66753
27.58267
26.93125
26.75072
26.43627
31.21886

Supplementary Table 4. **Raw data and t-tests comparing enamel hardness and elastic modulus along unpigmented and pigmented regions via nanoindentation in an *Alligator* tooth.** See Supplementary Figure 22 for locations of indents on tooth specimen.

**Alligator mississippiensis "Tooth 2" Hardness (GPa)**	
Not pigmented	Pigmented
3.775783	3.412174
3.364188	3.391806
3.48779	3.742697
3.056019	3.507157
2.58483	3.901373
3.144317	3.091216
3.763964	3.995661
2.470025	4.289471
3.469831	4.158713
2.986613	3.354019
3.736377	3.293483
3.55183	4.135276
3.018863	4.319894
2.958494	4.119581
3.45298	3.618454
4.118285	3.070825
3.54781	3.749877
3.387004	2.967326
3.252431	5.631001
4.053631	4.176532
3.334033	3.110942
3.461422	4.527254
3.405657	3.398391
3.788918	3.633499
3.16845	3.536072
3.685947	3.63776
3.158218	3.374321
3.666864	3.872065
3.296798	3.490606
3.188664	2.742493
4.601419	3.453633
3.147454	2.682982
3.159827	3.840887
2.945368	4.74339
3.022433	3.440588
3.167325	3.840911
3.241405	
3.219827	

t-Test: Two-Sample Assuming Unequal Variances

	Not pigmented	Pigmented
Mean	3.364239316	3.70145361
Variance	0.164721861	0.33571822
Observations	38	36
Hypothesized Mean Difference	0	
df	62	
t Stat	-2.885200425	
P(T<=t) one-tail	0.002687198	
t Critical one-tail	1.669804163	
P(T<=t) two-tail	0.005374396	
t Critical two-tail	1.998971517	

Cohen's d= 1.27319

Alligator mississippiensis "Tooth 2" Reduced Elastic Modulus (GPa)

Not pigmented	Pigmented
55.026182	51.408039
44.935707	53.550896
53.869358	54.530241
50.964051	50.164652
54.925842	54.739292
53.600073	53.121865
48.555829	56.551276
50.827675	60.089716
54.197168	54.61334
60.703161	51.965117
52.157949	51.2935
52.063885	56.090115
54.525315	50.96735
55.834203	56.595126
52.105861	53.197797
50.902528	42.757729
51.555696	49.591451
49.978844	46.125951
46.902866	61.33941
50.301403	51.384807
54.746308	46.439641
49.78486	54.03637
47.456246	47.450112
48.096927	49.199922
45.562022	46.354577
48.766688	48.913137
45.305957	47.057469
49.511884	48.864278
46.635941	47.224129

49.527946	43.411581
54.89623	45.698016
43.035233	40.408733
45.229968	49.678799
43.648261	55.72518
43.715317	48.508988
45.691991	48.556674
46.842171	
47.267549	

t-Test: Two-Sample Assuming Equal Variances

	Not pigmented	*Pigmented*
**Mean**	**49.99092355**	**50.7668132**
**Variance**	**16.71703991**	**21.9120228**
**Observations**	**38**	**36**
**Pooled Variance**	**19.2423788**	
**Hypothesized Mean Difference**	**0**	
**df**	**72**	
**t Stat**	**-0.760497479**	
**P(T<=t) one-tail**	**0.224720303**	
**t Critical one-tail**	**1.666293696**	
**P(T<=t) two-tail**	**0.449440605**	
**t Critical two-tail**	**1.993463567**	

Cohen's d= 0.177809

Supplementary Table 5: **Experimental parameters and data acquisition parameters used for LA-ICP-MS imaging.**

	Tooth samples	NIST 612 Scans
**Teledyne Photon Machines Iridia**		
Energy density (J cm^{-2})	2	3
Repetition rate (Hz)	333	100
Scan speed (μm s^{-1})	116.5	
Beam waist diameter (μm)	5 (square)	35 (square)
Scanning Mode	Fixed Dosage	Fixed Dosage
Scanning Direction	Uni-directional	Uni-directional
Effective Dosage (shots per position)	10	10
Helium carrier gas flow rate (L min^{-1})	0.3	0.3
Washout (ms)	30	30
**Thermo Fisher Scientific iCAP TQ ICP-MS**		
RF power (W)	1550	1550
Ar plasma gas flow rate (L min^{-1})	14	14
Ar auxiliary gas flow rate (L min^{-1})	0.8	0.8
Nebuliser gas flow rate (L min^{-1})	1.03	1.03
CR gas flow rate (L min^{-1})	0.16 (O_2)	0.16 (O_2)
ICP-MS mode	TQ	TQ
Acquired m/z ratios (amu)	^{24}Mg, $^{44}Ca^{16}O$, ^{56}Fe, ^{66}Zn, $^{88}Sr^{16}O$, $^{89}Y^{16}O$, $^{138}Ba^{16}O$	
Respective dwell times (ms)	1, 1, 1, 14.8, 1, 1, 1	
Total scan cycle time (ms)		30
