# Supplementary material for: Iron-coated Komodo dragon teeth and the complex dental enamel of carnivorous reptiles
Source: Nat Ecol Evol. 2024 Jul 24;8(9):1711–22. doi: 10.1038/s41559-024-02477-7 (PMC11383799; doi:10.1038/s41559-024-02477-7)
Supplement: Supplementary file 2 — Reporting Summary [file 41559_2024_2477_MOESM2_ESM.pdf]

nature portfolio

Corresponding author(s): Aaron R. H. LeBlanc

Last updated by author(s): 2024/03/23

Reporting Summary

Nature Portfolio wishes to improve the reproducibility of the work that we publish. This form provides structure for consistency and transparency in reporting. For further information on Nature Portfolio policies, see our Editorial Policies and the Editorial Policy Checklist.

Statistics

For all statistical analyses, confirm that the following items are present in the figure legend, table legend, main text, or Methods section.

n/a	Confirmed	
☐	☒	The exact sample size (*n*) for each experimental group/condition, given as a discrete number and unit of measurement
☐	☒	A statement on whether measurements were taken from distinct samples or whether the same sample was measured repeatedly
☐	☒	The statistical test(s) used AND whether they are one- or two-sided<br>*Only common tests should be described solely by name; describe more complex techniques in the Methods section.*
☐	☒	A description of all covariates tested
☒	☐	A description of any assumptions or corrections, such as tests of normality and adjustment for multiple comparisons
☐	☒	A full description of the statistical parameters including central tendency (e.g. means) or other basic estimates (e.g. regression coefficient) AND variation (e.g. standard deviation) or associated estimates of uncertainty (e.g. confidence intervals)
☐	☒	For null hypothesis testing, the test statistic (e.g. *F*, *t*, *r*) with confidence intervals, effect sizes, degrees of freedom and *P* value noted<br>*Give P values as exact values whenever suitable.*
☒	☐	For Bayesian analysis, information on the choice of priors and Markov chain Monte Carlo settings
☒	☐	For hierarchical and complex designs, identification of the appropriate level for tests and full reporting of outcomes
☐	☒	Estimates of effect sizes (e.g. Cohen's *d*, Pearson's *r*), indicating how they were calculated

Our web collection on statistics for biologists contains articles on many of the points above.

Software and code

Policy information about availability of computer code

Data collection: *Provide a description of all commercial, open source and custom code used to collect the data in this study, specifying the version used OR state that no software was used.*

Data analysis: Python v.3.7.4.; PyMCA v.5.6.3.; Microsoft Excel; DAWN v.2.27.0

For manuscripts utilizing custom algorithms or software that are central to the research but not yet described in published literature, software must be made available to editors and reviewers. We strongly encourage code deposition in a community repository (e.g. GitHub). See the Nature Portfolio guidelines for submitting code & software for further information.

Data

Policy information about availability of data

All manuscripts must include a data availability statement. This statement should provide the following information, where applicable:

- Accession codes, unique identifiers, or web links for publicly available datasets
- A description of any restrictions on data availability
- For clinical datasets or third party data, please ensure that the statement adheres to our policy

All data, measurements, and images are either in the manuscript, extended data, or supplementary information. For raw synchrotron XRF and XRD datasets, these are made publicly available through the relevant synchrotron facility three years after each experiment under a CC-BY-4 license.

Research involving human participants, their data, or biological material

Policy information about studies with human participants or human data. See also policy information about sex, gender (identity/presentation), and sexual orientation and race, ethnicity and racism.

Reporting on sex and gender	N/A
Reporting on race, ethnicity, or other socially relevant groupings	N/A
Population characteristics	N/A
Recruitment	N/A
Ethics oversight	N/A

Note that full information on the approval of the study protocol must also be provided in the manuscript.

Field-specific reporting

Please select the one below that is the best fit for your research. If you are not sure, read the appropriate sections before making your selection.

☐ Life sciences ☐ Behavioural & social sciences ☒ Ecological, evolutionary & environmental sciences

For a reference copy of the document with all sections, see nature.com/documents/nr-reporting-summary-flat.pdf

Ecological, evolutionary & environmental sciences study design

All studies must disclose on these points even when the disclosure is negative.

Study description

Several teeth of the Komodo dragon (Varanus komodoensis) were subjected to destructive analyses and used to characterize the structure and chemistry of the enamel along their tooth serrations. Teeth were embedded in resin, sectioned, polished, imaged, and prepared for several analytical techniques. Scanning Electron Microscopy (SEM), Energy Dispersal Spectroscopy (EDS), Focused Ion Beam (FIB) milling, Transmission Electron Microscopy (TEM), Synchrotron-based X-Ray MicroFluorescence (S-XRF), and Laser Ablation Mass Spectrometry (LA-MS) techniques were used to examine the structure of the enamel as well as its elemental composition. Element counts are recorded in the supplementary information of the manuscript for S-XRF and LA-MS and highlight the presence of zinc- and iron-coated serrations in V. komodoensis, a feature that is usually associated with increased wear-resistance in mammals.

We then examined the teeth of four species of extant crocodylian for evidence of iron-coated cutting edges along their teeth to assess the possibility that archosaurs also possess this unusual adaptation. For scouting this, we used a non-destructive technique (Laser-Stimulated Fluorescence-LSF) to determine that several species of extant crocodylian had different elemental concentrations along the cutting edges of their teeth. We then subjected these teeth to S-XRF and LA-MS to determine that iron (and zinc) were similarly present along their cutting edges, similar to V. komodoensis. The same characterization techniques were also used on a small sample of fossil crocodylian teeth from the Late Cretaceous Dinosaur Provincial Park locality of southern Alberta (Canada) to determine the effect of fossilization on our ability to detect iron layers in crocodylian teeth. We then conducted the same techniques on a sample of fossil tyrannosaurid and dromaeosaurid teeth from the same locality and non-destructive LSF imaging was conducted on a larger sample of museum specimens of other theropod dinosaur teeth. These findings demonstrated that fossilized crocodylian and theropod teeth show anomalous iron and zinc signatures throughout their dental tissues, making it nearly impossible to assess wither similarly-shaped theropod teeth possessed the iron-coated serrations we identified in modern Komodo dragons.

We then conducted Synchrotron-based X-Ray MicroDiffraction (S-XRD) on a sample of the tyrannosaurid teeth to quantify changed in enamel crystal structure along the serrations compared with other regions of the teeth to determine if theropod dinosaurs used alternative strategies for increasing the wear resistance of the cutting edges of their teeth. These results, along with SEM observations, demonstrated that tyrannosaurid dinosaurs in particular evolved a complex form of enamel that is elsewhere only seen in specialized herbivorous dinosaurs with grinding teeth, suggesting that this is a microfracture-mitigating adaptation within the serration enamel of tyrannosaurids.

To assess the mechanical properties of extant and fossil reptile enamels, nanomechanical testing was undertaken at Imperial College London.

Research sample

Komodo dragon (Varanus komodoensis) teeth were collected from various institutions (Museum of Life Sciences, KCL; University of the Sunshine Coast, Australia; Zoological Society of London, London Zoo), some of which were then sectioned and polished for various elemental and mechanical analyses. Of these, four teeth, (MoLS X263; J94036-1; J94036-2; J94036-5), derived from two individuals, were subjected to elemental analyses. We also surveyed museum collections, as well as a recently, ethically euthanized individual of V. komodoensis for the prevalence of orange, iron-coated serrations in the species. None of these were sampled destructively.

Shed teeth from four species of extant crocodylians (Tomistoma schlegelii, Osteolaemus tetrapsis, Crocodylus porosus, and Alligator mississippiensis) were used for elemental and mechanical testing.

	Two fossil crocodylian teeth, six tyrannosaurid teeth, and three dromaeosaurid teeth were loaned to O. Addison and A. LeBlanc for destructive analyses from the University of Alberta Laboratory for Vertebrate Palaeontology (UALVP). These were sectioned at various planes.
Sampling strategy	Sample sizes were based on availability of material at the respective institutions for destructive and non-destructive analyses.
Data collection	Light and electron microscopy images were collected by A. LeBlanc in the Centre for Oral, Clinical & Translational Sciences and the Centre for Ultrastructural Imaging (KCL) using in-house Keyence VHX7000 digital microscope and JEOL JCM-7000 SEM facilities. Additional SEM imaging and EDS analyses were conducted by Siyang Wang using a Zeiss Sigma 300 SEM at Imperial College London, while FIB milling and TEM-EDS were conducted by Catriona McGilvery at Imperial College London. STEM cross sections were prepared from the SEM sample using a ThermoFisher Scientific (TFS) Helios 5 CX with a Ga ion beam following standard milling procedures. TEM-EDS analysis was carried out at on a TFS Talos F200i equipped with an EDS detector. EDS analysis was performed at 200kV with the electron optics optimised for EDS analysis. TFS Velox software was used to process the data.<br><br>S-XRF, S-XRD, and Fe-XANES experiments and data collection were done by O. Addison, S. Sirovical, A. Morrell, and A. LeBlanc and the Diamond Light Source (UK) and the European Synchrotron Radiation Facility (France). Laser Ablation Inductively-Coupled Mass Spectrometry (LA-ICP-MS) was conducted on V. komodoensis and crocodylian teeth at the London Metallomics Facility. Additional laser ablation mass spectrometry of a Komodo dragon tooth using a Time-of-Flight system was conducted at the University of Southampton by Nu Instruments (credit: Phil Shaw) and appears in Supplementary Figure 3. All details are provided in the Materials and Methods, Extended Data, and Supplementary Information. Nanoindentation experiments were conducted by A. LeBlanc and D. Labonte using a Bruker Hysitron Ti950 with a Berkovitch indenter tip and the machine's proprietary software.<br><br>Laser Stimulated Fluorescence analysis was conducted by Michael Pittman and Thomas Kaye, and the images were processed using Adobe Photoshop.
Timing and spatial scale	Data collection for this project has been intermittent, between 2019-2024, including six synchrotron experiments at three beamlines across two facilities (UK and France), various museum collections visits in Canada, the UK, and Australia, nanomechanical testing experiments at Imperial College London, and electron microscopy at Imperial and King's Colleges.
Data exclusions	N/A
Reproducibility	Multiple teeth of Varanus komodoensis were subjected to elemental analyses to ensure repeatability of our findings. We also used multiple complementary elemental techniques (synchrotron X-ray microfluorescence; energy dispersive spectroscopy; laser ablation mass spectrometry) to ensure signals for elements of interest (e.g., iron, zinc, calcium) were consistent. We also conducted synhcrotron X-ray microfluorescence and laser ablation mass spectrometry for teeth of three species of crocodylian (Alligator mississippiensis, Crocodylus porosus, and Osteolaemus tetraspis). Where specimen-based observations were made (e.g., Supplementary information), we have recorded each specimen number and repository where these observations can be checked. For elemental and structural imaging experiments, all parameters are provided in the Materials and Methods, the Extended Data, and the Supplementary Information.<br><br>Synchrotron X-ray microdiffraction experiments on fossil tyrannosaurid teeth were conducted at two separate synchrotron facilities (Diamond Light Source, European Synchrotron Radiation Facility) and in two planes of section to ensure repeatability of our findings.<br><br>Scanning electron microscopy was conducted at two institutions (Imperial College London; Centre for Ultrastructural Imaging, KCL)
Randomization	No randomization processes were undertaken, due to the rarity of the extant and fossil reptile tooth samples. Samples were instead chosen based on their availability for destructive sampling.
Blinding	N/A

Did the study involve field work? ☐ Yes ☒ No

Reporting for specific materials, systems and methods

We require information from authors about some types of materials, experimental systems and methods used in many studies. Here, indicate whether each material, system or method listed is relevant to your study. If you are not sure if a list item applies to your research, read the appropriate section before selecting a response.

Materials & experimental systems

n/a	Involved in the study	
☒	☐	Antibodies
☒	☐	Eukaryotic cell lines
☐	☒	Palaeontology and archaeology
☐	☒	Animals and other organisms
☒	☐	Clinical data
☒	☐	Dual use research of concern
☒	☐	Plants

Methods

n/a	Involved in the study	
☒	☐	ChIP-seq
☒	☐	Flow cytometry
☒	☐	MRI-based neuroimaging

Palaeontology and Archaeology

Specimen provenance	All fossil dinosaur and crocodylian teeth used in this study were found in Dinosaur Provincial Park and are accessioned in the University of Alberta Laboratory for Vertebrate Palaeontology (UALVP). Specimens were loaned to O. Addison and A. LeBlanc for destructive analyses under loan numbers UALVP L2020-02 and UALVP L2023-06.
Specimen deposition	University of Alberta, Edmonton Canada.
Dating methods	N/A

☐ Tick this box to confirm that the raw and calibrated dates are available in the paper or in Supplementary Information.

Ethics oversight	University of Alberta Museums approved the fossil loans and destructive sampling requests to O. Addison and A. LeBlanc (UALVP L2020-02 and UALVP L2023-06.

Note that full information on the approval of the study protocol must also be provided in the manuscript.

Animals and other research organisms

Policy information about studies involving animals; ARRIVE guidelines recommended for reporting animal research, and Sex and Gender in Research

Laboratory animals	N/A
Wild animals	Shed teeth from four species of extant crocodylians were collected from the enclosures of live animals in the care of the Crocodiles of the World Conservation and Education Centre in Brize Norton, United Kingdom. Fluid-preserved Komodo dragon specimen was derived from the lower jaw of an ethically euthanized individual that was under the care of the ZSL London Zoo. The animal was not euthanized for this research. Applications for research on this individual from the London Zoo were submitted and approved by the ZSL.
Reporting on sex	N/A- we only had sex determination for the male V. komodoensis from the London Zoo. All remaining reptile teeth were either shed or from museum specimens for which sex was not recorded.
Field-collected samples	N/A
Ethics oversight	Loan of shed crocodylian teeth was approved by the Head of Education at Crocodiles of the World (Colin Stevenson). Loan of the lower jaw of Varanus komodoensis from the London Zoo was approved by Lewis Rowden (Zoo Research Officer, Zoological Society of London). This individual had to be ethically euthanized by Zoo veterinarians for unrelated reasons.

Note that full information on the approval of the study protocol must also be provided in the manuscript.
